# Phylogeography of *Pennella* (Copepoda: Siphonostomatoida: Pennellidae) indicates interoceanic dispersal mediated by cetacean and fish hosts

**DOI:** 10.1017/S0031182025000101

**Published:** 2025-02

**Authors:** Sofía Ten, Rachel Vanessa Pool, Juan Antonio Raga, Andrew D. Sweet, Francisco Javier Aznar

**Affiliations:** 1 Marine Zoology Unit, Cavanilles Institute of Biodiversity and Evolutionary Biology, University of Valencia, Paterna, Valencia, Spain; 2 Department of Biological Sciences, Arkansas State University, Jonesboro, AR, USA

**Keywords:** cetacean, dispersal, fish, genetic structure, parasitic copepod, phylogeny, taxonomy

## Abstract

Copepods of the genus *Pennella* parasitize a wide range of marine animals, including cetaceans, teleosts, and cephalopods worldwide. Their taxonomy is unclear, as there is incongruence between morphological and genetic data and incomplete species coverage. This study provides new morphological and genetic (COI) data from 23 specimens of *Pennella* cf. *filosa* (syn. *P. balaenoptera*) from western Mediterranean whales and a swordfish. First, their position in the phylogeny of *Pennella* was assessed and species delimitation revisited using all available *Pennella* COI sequences (*n* = 189), obtained from Mediterranean and north Pacific specimens from 18 host species (including multiple cetaceans and teleosts). Second, it was investigated whether the geographic location, degree of host vagility, or host taxonomic identity help explain genetic differentiation. Five distinct haplotype groups with varying genetic divergence were distinguished. Although the presence of sibling species cannot be ruled out, species delimitation methods could not find interspecific genetic differences, leaving the taxonomy of the genus unresolved. The observed genetic differentiation could not be attributed to geography or host type. This suggests that members of the genus *Pennella* show low specificity for definitive hosts and interoceanic dispersal mediated by some vagile definitive hosts. The use of more genetic markers for addressing these questions in the future is encouraged.

## Graphical abstract

**Figure:**
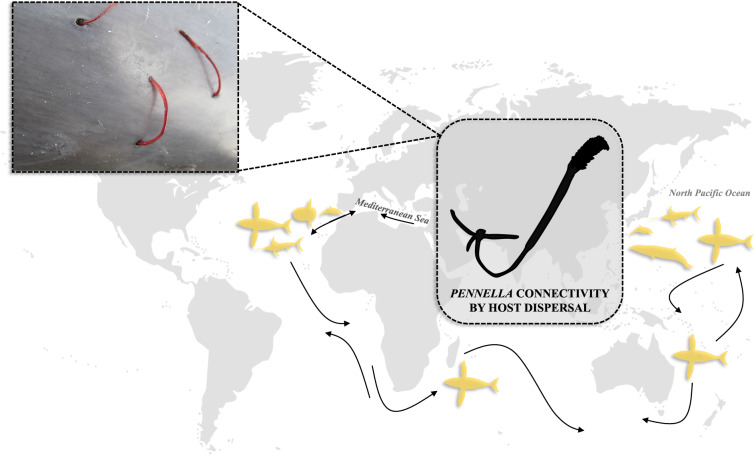

## Introduction

Copepods of the genus *Pennella* Oken, 1815 (Order Siphonostomatoida) are circumglobal marine parasites that infect a broad range of animals, including cetaceans, teleosts, and cephalopods. Their complete life cycle remains unknown. In the sister genus *Lernaeenicus* Lesueur, 1824, there is a *ca.* 2-day planktonic phase with two naupliar stages. The planktonic phase is followed by a copepodid stage that infects an intermediate host, on which the parasite undergoes three chalimus stages before mating, then the inseminated female seeks a definitive host on which it attaches, metamorphoses, and releases over 1,000 eggs (Whitfield et al., 1988; Izawa, 2019). It is believed that *Pennella* spp. use flatfish or cephalopods as intermediate hosts for mating, and fertilized females then infect the definitive host (i.e. a cetacean or teleost). Fertilized females remain partly embedded in the host’s skin and blubber and feed on body fluids while leaving their trunk, abdomen, and gills hanging outside (Turner, 1905; Kabata, 1979; Anstensrud, 1992; Arroyo et al., 2002; Boxshall et al., 2005). Heavy infections of *Pennella* spp. have been associated with increased mortality in small fish (Suyama et al., 2021a), whereas in cetaceans they can indicate poor host health (Vecchione and Aznar, 2014; Chaieb et al., 2024).

The taxonomy of the genus *Pennella* has traditionally been based on female morphology (e.g. number of antennae or cephalothorax shape) and the identity of the definitive hosts. Recent efforts using these two criteria have progressively reduced the number of species from dozens to nine: *P. filosa* (Linnaeus, 1758), *P. balaenoptera* Koren & Danielssen, 1877, *P. sagitta* Linnaeus, 1758, *P. benzi* Hogans, 2017, *P. instructa* Wilson, 1917, *P. makaira* Hogans, 1988, *P. exocoeti* (Holten, 1802), *P. diodontis* Oken, 1816, and *P. hawaiiensis* Kazachenko & Kurochkin, 1974 (Hogans, 2017 and references therein). Recently, Suyama et al. (2021b) examined over one hundred specimens of north Pacific and Mediterranean origin and proposed a total of 2–3 *Pennella* species complexes based on morphological traits. First, the *P. sagitta* species complex – also named Group I – is composed of fish parasites that have large branched antennary processes and a total length ≤90 mm (Suyama et al., 2019; 2021b). Second, the *P. filosa* complex is composed of large-sized pennellids, with a total length >80 mm, that lack branched antennary processes (i.e. Groups II and III; Suyama et al., 2021b); this complex includes *P. filosa, P. benzi* and *P. instructa*, all of which infect teleosts (Fraija-Fernández et al., 2018; Suyama et al., 2021b), as well as *P. balaenoptera*, which has been documented on over 20 cetacean species and once on a pinniped (Dailey et al., 2002; Ten et al., 2022). In fact, Fraija-Fernández et al. (2018) previously suggested that *P. balaenoptera* could be synonymized with *P. filosa* based on morphological and molecular evidence. Lastly, the small-sized *P. makaira*, parasitic on swordfish, could not be assigned to any of these two complexes due to insufficient morphological data and some confusing traits (Suyama et al., 2021b).

Morphology, however, may be of limited use for species delimitation in the genus *Pennella* since specimens show great morphological plasticity depending on ontogenetic development (e.g. parasites recently attached to the definitive host lack antennae), and on the host and attachment site (Kabata, 1979; Hogans, 1987). Given this high morphological polymorphism and the convergent traits shared among the Pennellidae (e.g. Castro-Romero et al., 2016; Yumura et al., 2022), molecular techniques become particularly relevant for studying the taxonomy of this group. However, the available evidence (see Suyama et al., 2021b) indicates a clear incongruence between morphological and molecular data, suggesting that species delimitation within the genus is still challenging.

Also, host identity is no longer a robust criterion for species delimitation. The proposed species complex *P. filosa* stands out for its very low host specificity and it shares a host species (i.e. the ocean sunfish) with the putative species complex *P. sagitta*. These pennellids are unique among metazoan parasites as they are able to parasitize a great diversity of both fish (e.g. swordfish, sunfish, or pufferfish) and cetaceans, including whales and dolphins, from all oceans. The low host specificity and global distribution of *Pennella* cf. *filosa* (and, potentially, of other *Pennella* spp.) suggest that the degree of population structure is probably low, but this hypothesis has never been addressed.

The present study investigates the phylogeography and host specificity of members of *Pennella* based on data from the cytochrome c oxidase subunit 1 (COI) mitochondrial gene. COI has been, by far, the most commonly used DNA barcoding marker for siphonostomatoid copepods, not only in phylogenetic and phylogeographic studies, but also for species delimitation and for investigating genetic differences between hosts (e.g. Boulding et al., 2009; Dippenaar, 2009; Dippenaar et al., 2010; Mangena et al., 2014; Morales-Serna et al., 2014; Skern-Mauritzen et al., 2014; Castro-Romero et al., 2016). We first provided new morphological and genetic (i.e. COI) data from a number of specimens of *Pennella* cf. *filosa* (syn. *P. balaenoptera*) collected in the western Mediterranean, then we assessed their position in the phylogeny of *Pennella* and revisited species delimitation after Suyama et al. (2021b). Secondly, for the phylogeographic and host specificity analyses we used all available sequences of *Pennella* spp. since the taxonomy of the genus was unresolved. In particular, we examined the genetic differentiation between specimens collected in the Mediterranean Sea and north Pacific Ocean, and explored other factors that might contribute to genetic structuring, i.e. host identity and degree of host vagility between oceanic basins.

## Materials and methods

### Data collection

A total of 60 parasites tentatively identified as *Pennella filosa* (syn. *P. balaenoptera*) – see Results – were obtained from six dead whales stranded along the coast of Spain, ranging from 40°31.50’N, 0°31.00’E to 37°50.70’N, 1°37.50’W, and a dead swordfish found in Castellón, 39°58.17’N 0°00.84’E (Table 1). Permission and funding to collect stranded animals were given by the Wildlife Service of the Valencian Regional Government, Spain. Some parasites lost the cephalothorax during collection, but they could all be identified following morphological criteria (e.g. Abaunza et al., 2001; Hogans, 2017).

**Table 1.** Studied specimens of *Pennella* cf. *filosa* (syn. *P. balaenoptera*) from six stranded whales and a swordfish, all from the western Mediterranean. The number (*n*) of specimens examined morphologically and sequenced is indicated

Host species	Host total length (cm)	Stranding year	n morphology	n molecular	Accession number(s)
Fin whale, *Balaenoptera physalus*	1230	1982	1	1	[PP908436]
	982	2011	3	3	[PP908428, PP908432, PP908434]
	594	2020	43	5	[PP908439-PP908443]
	1450	2021	2	2	[PP908445, PP908446]
Humpback whale, *Megaptera novaeangliae*	832	2019	3	3	[PP908426,PP908430, PP908444]
	1560	2022	7	8	[PP908425, PP908427, PP908429, PP908431, PP908433, PP908435, PP908437, PP908438]
Swordfish, *Xiphias gladius*	NA	2019	1	1	[PP908447]

A subset of 23 specimens was selected for molecular identification and phylogeographic analyses. The selected subs*et al*lowed for the investigation of differentiation between specimens from different hosts and, in some cases, between those collected from the same host individual (Table 1). We used the DNeasy Blood & Tissue Kit (QIAGEN) for DNA extraction from *ca.* 2 mm^3^ of tissue from the trunk or neck of each specimen. Partial mitochondrial cytochrome c oxidase subunit I (COI) was amplified with a *Pennella*-specific primer pair designed by Suyama et al. (2020): HijikiCOI-F (5′-GGATATTGGRACTTTGTACTTATTAAG-3′) and HijikiCOI-R (5′-AAAAATCAAAATAAATGCTGG-3′), each at a concentration of 5 pmol/μl. PCR reaction mixtures had a final volume of 20 μl, with 2 μl DNA, 4.8 μl molecular grade water, 1.6 μl of each primer, and 10 μl MyFi™ DNA Polymerase (BioLine, Meridian Life Science Inc., Taunton, MA, USA). Thermocycling profiles for gene amplification were as follows: initial denaturation at 94°C for 5 min, 38 cycles of 94°C for 45 s, 50°C for 45 s, 72°C for 80 s; and a final extension at 72°C for 7 min. Positive and negative (no DNA) controls were used in each PCR.

Aliquots of 2 μl of each amplicon were mixed with 2 μl of loading dye and run on an agarose gel (1% gel; 0.4 g agar powder and 40 ml TE buffer) stained with 1 μl GelRed® Nucleic Acid Gel Stain (Biotium, Hayward, CA, USA) for electrophoresis. The bands were visualized and photographed using an ultraviolet light hood. Amplicons were purified with the Nucleospin® PCR and Gel Purification Clean-up kit (Macherey-Nagel, Düren, Germany) and were sent to Macrogen Europe (Amsterdam, Netherlands) for sequencing with the HijikiCOI primer pair. Sequence identity was verified using the Basic Local Alignment Search Tool (BLAST; https://blast.ncbi.nlm.nih.gov/Blast.cgi). All 23 sequences were uploaded to GenBank (see accession numbers in Table 1).

The 23 COI sequences, along with the other 166 available COI sequences of *Pennella* spp. in GenBank (searched until January 2024; including those in Fraija-Fernández et al., 2018; Suyama et al., 2021b; Table S1) and 10 outgroups (see Fraija-Fernández et al., 2018), were aligned using MUSCLE within Geneious Prime 2024.0 (https://www.geneious.com) with default settings. The alignment length was 447 bp. We found no evidence that any of the 189 *Pennella* sequences were nuclear-mitochondrial DNA segments (NUMTs), i.e. mitochondrial DNA fragments inserted into the nuclear genome as non-functional pseudogenes (Porter and Hajibabaei, 2021; Xue et al., 2023). For this purpose, we checked for the presence of indels or stop codons and double peaks in the Sanger chromatogram (for those available), and compared GC content and translation to that of a complete COI gene of *Pennella* sp. (GenBank accession number: ON161759). Details of all the aligned sequences, including host identity and morphological identification, are provided in Table S1.

### Phylogenetic position and genetic structure

The resulting alignment of 189 sequences was used to investigate phylogenetic relationships between the 23 new Mediterranean specimens and those from the two species complexes proposed by Suyama et al. (2021b), i.e. *P. sagitta* and *P. filosa*, and also with the unclassified sequences. Phylogenetic analyses were performed with the General Time Reversible (GTR) nucleotide substitution model with a Gamma rate of variation, selected according to the Akaike Information Criterion (AIC) by jModelTest (Guindon and Gascuel, 2003; Darriba et al., 2012) on the CIPRES Science Gateway server (Miller et al., 2010). A Bayesian analysis was performed in MrBayes 3.2.7 (Huelsenbeck et al., 2001), and posterior probability distributions were generated by four simultaneously running Markov chains using 10 M generations. We considered that convergence was achieved if the potential scale reduction factor (PSRF) ∼ 1 and the average standard deviation of split frequencies (ASDSF) ∼ 0.01 (in MrBayes). The stationarity of the runs was assessed by plotting MCMC generations versus the log-likelihood values of the data in Tracer v1.7.2 (Rambaut and Drummond, 2009). Also, an effective sample size (ESS) >200 for each parameter was considered acceptable; this was also checked in Tracer. A total of 25% of the trees were discarded as burn-in. For the Maximum Likelihood (ML) analysis, conducted in RAxML (Stamatakis, 2014), we set the number of bootstrap replications to 1 M. Tree topologies of the Bayesian and ML trees were checked for congruence using the program FigTree v.1.4.4 (Rambaut, 2010).

We also examined potential drivers of genetic differentiation, i.e. the (1) geographic region of the sample (north Pacific *vs.* western Mediterranean), (2) degree of dispersal of the hosts (samples from host species with interoceanic dispersal and genetic exchange *vs.* hosts with a smaller distribution range within an ocean basin; see Table S2), and (3) host taxon (i.e. members of the classes Teleostei and Mammalia, and between teleost orders and cetacean superfamilies). Note that all available sequences from the Mediterranean Sea were identified as *P. filosa* (syn. *P. balaenoptera*). To this end, we firstly built a parsimony haplotype network (TCS) of the 189 sequences with PopART (Clement et al., 2002; Leigh and Bryant, 2015). This approach was considered pertinent due to the nature of our dataset, i.e. low genetic divergence (see Suyama et al., 2021b) and the observed reticulate relationships (see Results and, e.g. Bandelt et al., 1999; Clement et al., 2000). In any case, TCS topology was very similar to that of Median-joining (MJN) and Minimum-spanning (MSN) networks, also built with PopART. A reticulate network based on distance corrected with the Kimura 2-parameter (K2P) and uncorrected p-distances was generated with the NeighborN*et al*gorithm in SplitsTree v.4.19.2 (Bryant and Huson, 2023).

Secondly, analyses of molecular variance (AMOVA) were conducted using the software ARLEQUIN version 3.5.2.2 (Excoffier and Lischer, 2010) to test for significant differentiation among (1) geographic regions, (2) types of host dispersal, (3) host taxa (i.e. teleosts *vs.* cetaceans, including mysticetes and odontocetes), and also (4) the five haplogroups identified by the parsimony haplotype network (see Results). To test for population structure, pairwise differences in fixation index (F_ST_) were also calculated for the five haplotype groups and significance was evaluated with 10,000 permutations. Nucleotide evolutionary divergence between (and within) groups was estimated using the K2P model in MEGA11. The rate variation among sites was modeled with a gamma distribution, following AIC-based model selection from the jModel test (see above), and the number of bootstrap replicates was set to 1,000. Nucleotide divergence among host taxa was also estimated separately for western Mediterranean and north Pacific samples to account for the possible effect of geographic region.

### Species delimitation

With only COI sequences available, we attempted to identify potentially differentiated genetic lineages within *Pennella* using a multi-step (exploratory) approach with all 189 sequences. First, we used the distance-based approaches ABGD (Automatic Barcode Gap Discovery; Puillandre et al., 2012) and ASAP (Assemble Species by Automatic Partitioning; Puillandre et al., 2021). For ABGD, we set the intraspecific prior divergence between 0.001 and 0.1. Both methods were run twice with simple distance and K2P evolution models, respectively, on their web interfaces (https://bioinfo.mnhn.fr/abi/public/abgd/ and https://bioinfo.mnhn.fr/abi/public/asap/).

Second, we used the Bayesian implementation of Poisson Tree Processes (bPTP; http://species.h-its.org/ptp/; Zhang et al., 2013). PTP is considered to efficiently deal with single-locus data (Tang et al., 2014), and it was preferred over GMYC models (generalized mixed Yule-coalescent; Pons et al., 2006), another tree-based approach, because it does not require time calibration (an error-prone process; Zhang et al., 2013; Dumas et al., 2015). We used the ML tree without outgroups (created following the methodology above) as the input to improve the delimitation results (Zhang et al., 2013). The number of MCMC generations was set to 200,000 and the other parameters were left as default. We checked for PTP convergence by visual inspection of the likelihood plot (Zhang et al., 2013).

## Results

### Morphological identification of new Mediterranean specimens

**Figure 1.**
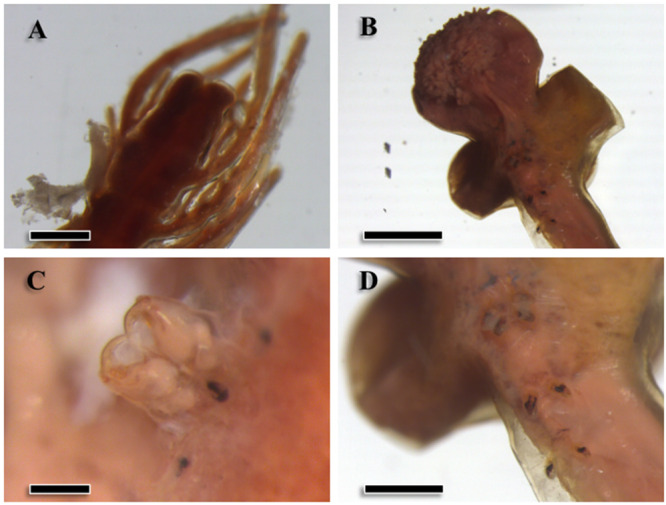
Morphological traits of specimens of *Pennella balaenoptera* from a fin whale, *Balaenoptera physalus*, stranded in the western Mediterranean. A, terminal region of the abdomen (scale bar: 0.5 mm); B, cephalothorax (2 mm); C, secondary antennae in the antennary region (0.2 mm); D, detail of the swimming legs (1 mm).

Specimens were large pennellids (see Hogans, 2017), with minimum estimated total lengths of approximately 100 mm. In five intact specimens, total lengths ranged from 113.0 to 275.0 mm (mean ± SD: 150.3 ± 33.5 mm). The abdomen (mean length: 17.5 ± 9.6 mm, *n* = 16; Figure 1A) was dark brown and exhibited numerous abdominal plumes expanding outwards; the trunk (mean length: 19.9 ± 12.7 mm, *n* = 48; mean maximum width: 1.8 ± 1.7 mm, *n* = 40) was similar in color and presented a striated contour. Eight larger specimens (estimated mean total length: 219.0 ± 48.0 mm) presented egg strings that emerged from the base of the abdomen. The neck was thin and long (mean length: 88.8 ± 7.7 mm, *n* = 14) and paler in color. The globose head (mean length: 4.9 ± 1.3 mm and mean maximum width: 4.5 ± 1.5 mm; *n* = 6) was covered with small and numerous papillae (Figure 1B) and presented the antennary region on its dorsal side (Figure 1C). The adjacent thoracic region presented two lateral holdfast horns (mean length: 17.3 ± 8.5 mm, *n* = 6; Figure 1B), sometimes also a smaller dorsal horn (8.3 ± 6.4 mm, *n* = 5), and four pairs of swimming legs ventrally (Figure 1D). Therefore, specimens were morphologically identified as *Pennella filosa*, syn. *P. balaenoptera* (*sensu* Abaunza et al., 2001; Hogans, 2017; Fraija-Fernández et al., 2018).

### Phylogenetic position and genetic structure

## Phylogenetic tree and haplotype network structure

**Figure 2.**
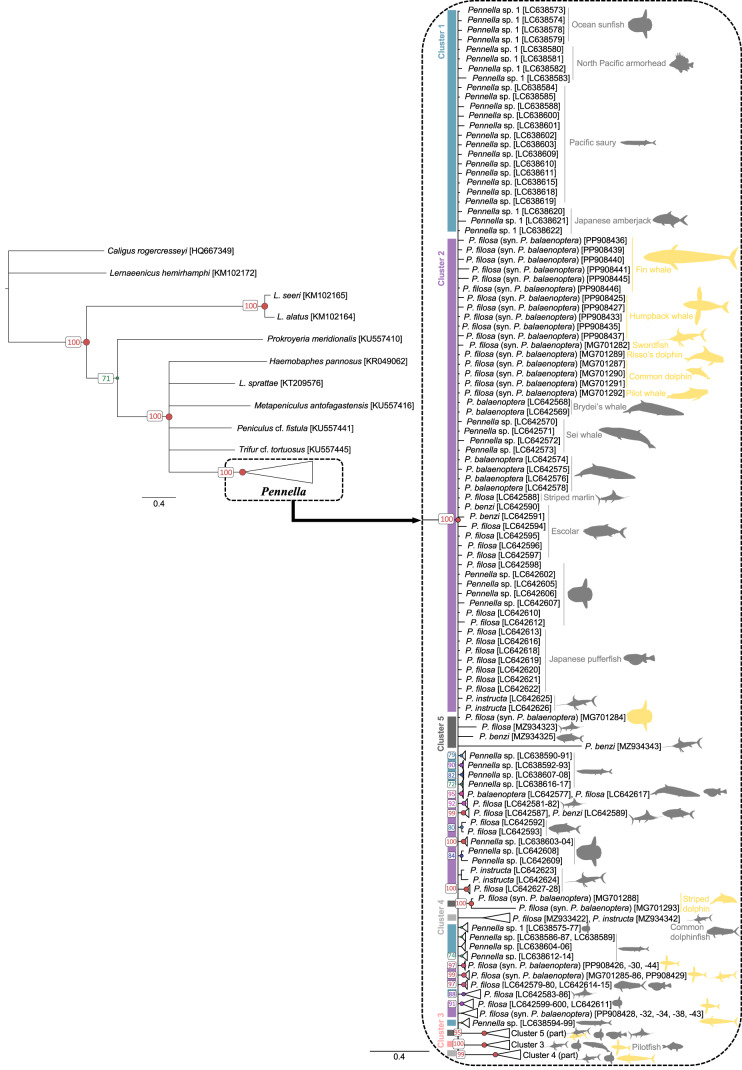
Bayesian inference phylogenetic tree based on COI sequences of 189 specimens of the genus *Pennella*. Host identity is indicated by icons, colored by geographic origin (grey, north Pacific; yellow, western Mediterranean). Support values for each node are expressed as posterior probabilities; values <70% are not shown. color bars and cluster numbers indicate the haplogroups from the haplotype network in Figure 3. Horizontal bars indicate evolutionary distance.

Phylogenetic trees grouped all *Pennella* sequences in a clade, separated from the outgroups with >95% support. Bayesian (Figure 2) and ML (Fig. S1) topologies were very similar, but with much higher support values for Bayesian inference. This analysis reached both convergence and stationarity since PSRF = 1.001, ASDSF = 0.008, log-likelihood values fluctuated around a horizontal line, and ESS >300 for all parameters. The 23 newly obtained sequences were identified as *P. balaenoptera* with BLAST searches but were widespread on the phylogenetic tree (Figure 2), with some being more closely related to north Pacific than to other Mediterranean samples (see bottom nodes in Figure 2). Except for the sequence from the swordfish parasite, the other 22 samples are the first sequences from pennellids associated with humpback and fin whales.

**Figure 3.**
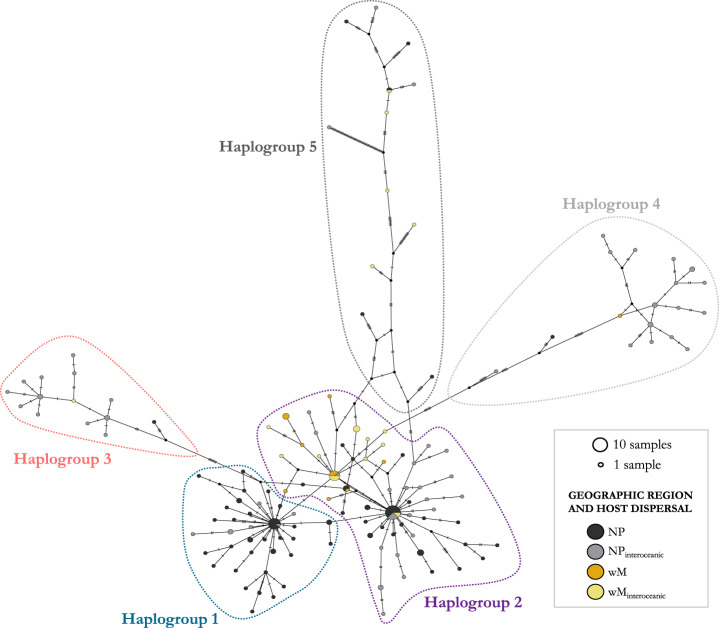
Parsimony haplotype network of COI sequences from *Pennella* spp. specimens. Haplotype frequency is proportional to circle area. Colors of the circles represent the geographic origin of the samples (black, gray: north Pacific; orange, yellow: western Mediterranean) and the degree of dispersal of the Hosts (gray, yellow: Host species with interoceanic connectivity; black, orange: Hosts with lower dispersal). Five major haplogroups were identified in the network (outlined areas); see the main text for details. Sequence identity and accession numbers can be found in Table S1.

The overall structure of the haplotype network was complex (Figure 3). A total of 145 COI haplotypes were found among the 189 sequences (Table S1). Suyama et al. (2021b) detected 126 haplotypes, so the 23 new *Pennella* sequences from the western Mediterranean represent 19 unique haplotypes (Table S1). The new specimens that did not represent unique haplotypes shared haplotypes with north Pacific specimens (i.e. the specimen from the swordfish, Xg5, and one each from a humpback and fin whale, Mn1 and Bp3 respectively; Table S1). At least one specimen from highly vagile host species (e.g. a humpback whale, swordfish, or sunfish; Table S2) was present when haplotypes were shared between parasites from the two geographic regions, except for haplotype XLVI, which includes pennellids from a Mediterranean fin whale and a north Pacific escolar (Table S1).

We identified 5 haplogroups on the network; Haplogroups 3–5 were separated by >7 mutational steps (i.e. nucleotide substitutions) from the center of the network, composed of the two more closely related Haplogroups 1 and 2 (Figure 3). Despite the low number of mutational steps between Haplogroups 1 and 2 (about 1; Figure 3), they were morphologically distinct. Samples in Haplogroup 1 were smaller (total length < 74 mm) and showed branched antennary processes on the cephalothorax (Group I in Suyama et al., 2021b, putatively the species complex *P. sagitta*), whereas those in Haplogroups 2 and 3 were larger and lacked branched processes (Figure 1; Groups II and III in Suyama et al., 2021b, putatively the *P. filosa* species complex). Samples in Haplogroups 4-5 had the same morphology as those from Haplogroups 2-3.

In both phylogenetic trees, sequences from Haplogroups 1 and 2 (Figure 3) were not differentiated and displayed a comb shape (Figures 2 and S1). Except for the 23 new Mediterranean samples, these sequences would correspond to samples from the species complex *P. sagitta* (Group I) and some of the complex *P. filosa* (Group II) *sensu* Suyama et al. (2021b). By contrast, Haplogroup 3 always constituted a monophyletic clade (with ≥98% support; Figure 2; also the monophyletic Group III, considered part of the *P. filosa* complex in Suyama et al., 2021b). The Bayesian tree suggested, with 99% support, that Haplogroup 4 belongs to a monophyletic clade, while the paraphyletic Haplogroup 5 may be subdivided into three genetic lineages (Figure 2). ML was not very informative for these two haplogroups as support values were extremely low (i.e. 0–3%; Fig. S1). Reticulate relationships were frequent between and within the five haplogroups (Fig. S2), with similar topologies between the reticulate networks based on K2P and uncorrected p-distances.

There were three relatively abundant haplotypes (shared by 7–13 samples) at the center of the network, i.e. one in Haplogroup 1 and two in Haplogroup 2. Two of these included mostly north Pacific samples and constituted star-like structures with most branches being short (generally <4 mutational steps, up to 6 in a few branches) (Figure 3). A large proportion of Mediterranean samples was clustered at the very center of the network, in Haplogroup 2 (see below), and were separated from those in Haplogroup 3 by at least 11 mutational steps. The number of steps within Haplogroup 3 was much lower (i.e. 1–6). Haplogroups 4 and 5 were connected to the central haplogroups through many mutational steps (i.e. 8–68 steps, from the closest to the most distant samples). Haplogroups 4 and 5 exhibited a much more ramified structure, with a broad range of mutational steps between samples (i.e. 1–49; Figure 3), and correspond to Group IV in Suyama et al. (2021b), which was considered a group of NUMTs. Branches with > 10 mutational steps belong to highly divergent haplotypes (see Feis et al., 2015). We found no evidence of poorer sequence quality in these two haplogroups (i.e. similar GC content, 26.9–37.8%; and percentage of base ambiguities, with all group means <0.07%).

## Geographic and host effect on genetic differentiation

Despite the aforementioned genetic structure within *Pennella*, the observed differentiation could not be associated with the geographic region where samples had been collected nor with host taxon or host dispersal pattern (i.e. interoceanic connectivity). The haplotype network highlighted some patterns that would go unnoticed by just looking at phylogenetic trees. Mediterranean samples were clustered at the center, although they were well represented throughout the network – except for Haplogroup 1, with only north Pacific samples – and shared haplotypes with north Pacific samples from Haplogroups 2 and 5, regardless of the degree of host dispersal (Figure 3). The proportion of hosts that conduct interoceanic movements was lower in Haplogroup 1 while larger in Haplogroups 3–5, although we failed to detect a significant effect (see below). Haplotype relationships were also decoupled from host identity or taxonomy; some specimens from different hosts were more closely related, and even shared haplotypes, than those co-occurring on the same host individual (e.g. parasites of Mn1; Table S1). In fact, genetic differentiation was detected at the infrapopulation level (i.e. within an individual host), with co-occurring parasites being assigned to two different haplogroups. This was reported in 10 hosts from 9 species, including mysticetes and teleosts from both geographic regions and dispersal types. One additional case involved the parasites of a north Pacific swordfish, grouped into three different haplogroups (Table S1).

The AMOVAs showed that most of the genetic variation (>85%) occurred within, rather than between, geographic regions and host types (Table 2). Mean nucleotide sequence divergence (% K2P ± standard error) between geographic regions (4.4 ± 5.5) or between host types (4.2 ± 1.9 between taxa, Table S3; 5.2 ± 0.7 for dispersal) was, in some cases, lower than within groups (i.e. 4.98% among north Pacific samples and 6.87% among samples from interoceanic hosts). The lower divergence between cetacean superfamilies than between cetaceans and teleosts could be an artifact of the high divergence among the teleosts (Table S3), observed in both geographic regions (Table S4). In contrast, there was significant genetic heterogeneity between the five haplogroups (Table 2).

**Table 2.** Results of the AMOVA among *Pennella* spp. sequences from two geographic regions (north Pacific and western Mediterranean), from hosts with a varying degree of vagility (interoceanic movement and gene exchange vs. smaller range within an ocean basin), and from five haplogroups inferred from previous analyses (see main text)

Source of variation	Sum of squares	Variance components	Percentage variation	*p*-value
*Geographic regions*				
Among populations	35.45	0.23	2.51	<0.00001
Within populations	3386.73	9.01	97.49	
Total	3422.17	9.24		
*Host dispersal*				
Among populations	255.30	1.31	13.45	<0.00001
Within populations	3166.87	8.42	86.55	
Total	3422.17	9.73		
*Host taxa*				
Among populations	79.10	0.45	4.82	<0.00001
Within populations	3343.07	8.92	95.18	
Total	3422.17	9.37		
*Haplogroups*				
Among populations	2252.56	8.42	72.80	<0.00001
Within populations	1169.62	3.14	27.20	
Total	3422.172	11.56		

Pairwise F_ST_ differences between haplogroups were all significant at the 95% significance level (*p* < 0.0001; Table 3). Nucleotide sequence divergence was low between Haplogroups 1 and 2 (K2P: 1.3%, and also lower F_ST_, i.e. 0.3), intermediate between both 1–2 and 3 (>4%), and very high for all combinations including Haplogroups 4 or 5 (>10%), matching the patterns in the phylogenetic trees (Figures 2 and S1) and networks (Figures 3 and S2). Haplogroup 5 was particularly diverse, with intra-haplogroup K2P ∼ 9% (*vs.* ≤3% for the rest; Table 3).

**Table 3.** Genetic diversity among 189 COI sequences of *Pennella* spp. from five haplogroups identified in a parsimony haplotype network. Values represent pairwise differences in F_ST_ (above diagonal) and mean nucleotide pairwise sequence divergence (% K2P ± standard error) between (below diagonal) and within (shaded values on diagonal) haplogroups. All F_ST_ differences are significant, with all nominal *p*-values <0.0001

	Haplogroup 1 (*N* = 50)	Haplogroup 2 (*N* = 89)	Haplogroup 3 (*N* = 16)	Haplogroup 4 (*N* = 21)	Haplogroup 5 (*N* = 13)
**1**	0.58 ± 0.10	0.34	0.84	0.89	0.74
**2**	1.27 ± 0.34	1.04 ± 0.18	0.76	0.86	0.73
**3**	4.38 ± 0.98	4.69 ± 1.00	0.91 ± 0.22	0.85	0.630
**4**	11.49 ± 1.94	11.06 ± 1.85	12.32 ± 2.03	2.23 ± 0.33	0.523
**5**	11.44 ± 1.61	11.19 ± 1.57	12.79 ± 1.77	10.70 ± 1.40	9.08 ± 1.05

### Species delimitation

Species delimitation methods did not find consistent evidence of interspecific differentiation between the 189 analyzed COI sequences, a matter that should be directly addressed with more markers (see Discussion). First, ABGD and ASAP show a lack of ‘barcode gap,’ i.e. the gap between intraspecific and interspecific variation. Second, bPTP had low support values at the internal nodes and showed a tendency toward over-splitting, with 37 ‘species’ detected.

## Discussion

The present phylogeny based on COI sequences supports that the genus *Pennella* constitutes a monophyletic group within the order Siphonostomatoida (Fraija-Fernández et al., 2018; Suyama et al., 2021b). All the specimens from this study – collected from western Mediterranean whales and a swordfish – were identified as *Pennella filosa* (syn. *P. balaenoptera*) following previous morphological and molecular criteria. These specimens were interspersed within the phylogeny of *Pennella*, which generally displayed a comb shape.

Based on distinct morphological traits, Suyama et al. (2021b) proposed that *Pennella* could be grouped into 2 species complexes – namely *P. sagitta* and *P. filosa* – and possibly a third species, *P. makaira*. When also considering molecular data, however, this taxonomic classification becomes controversial. First, there is clear incongruence between morphological and molecular data. Second, we failed to find significant genetic differentiation through species delimitation methods or by comparing sequence divergence between samples from the putative species complexes *P. sagitta* and *P. filosa* (Suyama et al., 2021b), which correspond to Haplogroups 1 and 2–3, respectively (see below). Interspecific nucleotide divergence is very variable within genera of siphonostomatoid copepods (generally 14.4–30.1%; Øines and Schram, 2008; Dippenaar et al., 2010; Muñoz et al., 2015; Lovy and Friend, 2020). There are also cases of sibling or cryptic species of siphonostomatoids with divergences 12–17% (Øines and Heuch, 2005; Øines and Schram, 2008; Dippenaar et al., 2010). The divergence values between Haplogroups 1–3 (i.e. 1.3–4.7%) in our study are therefore compatible with intraspecific variation. In contrast, the divergence between Haplogroups 1–3 and 4 or 5, and between Haplogroups 4 and 5, was larger (i.e. 10–12%), hence we cannot rule out the possibility of cryptic species – specimens in Haplogroups 2–3, 4, and 5 could represent three sibling species that share morphology (that of the *P. filosa* complex *sensu* Suyama et al., 2021b). In contrast, Haplogroups 1 and 2–3 could constitute two morphotypes of a single species – Castro-Romero et al. (2016) found very low genetic distance (0.95%) among morphologically different specimens of the pennellid *Peniculus* cf. fi*stula* (see also Lovy and Friend, 2020). Note, however, that this classification into 3 putative species was not supported by species delimitation methods. Also, Suyama et al. (2021b) stated that sequences in Haplogroups 4–5 (i.e. Group IV) were NUMTs, although we did not find conclusive evidence for this assumption.

Another possibility for the incongruence between morphological and molecular data could be incomplete lineage sorting since only a single molecular marker was available. This would mean that COI might not be the most suitable marker for species delimitation in *Pennella*, even if reliable for other siphonostomatids (e.g. Castro-Romero et al., 2016). Another example is the molecular marker ITS1, which failed to reveal the clades identified in the COI phylogeny (Suyama et al., 2021b). Furthermore, the high morphological polymorphism among the Pennellidae (Kabata, 1979; Hogans, 1987) hampers morphology-based inferences on species delimitation. Therefore, the taxonomy of *Pennella* remains in a state of flux and should be investigated in future studies by incorporating multiple loci (including at least one nuclear marker other than ITS1) to provide reliable measures of genetic differentiation. In the meantime, using species classification by Hogans (2017) or Suyama et al. (2021b) could be a practical approach for referring to specific morphologies, and the use of the qualifier ‘cf.’ (Latin: *conferre*) before the species name is recommended.

Phylogenetic networks are useful for displaying relationships that may not be bifurcating, while accounting for the effect of gene flow (Blair and Ané, 2020). We identified 5 haplogroups in a COI-based haplotype network, and they were all linked by reticulate relationships. The structure of the network, with a few shared haplotypes and relatively low levels of nucleotide diversity (i.e. ≤2% for Haplogroups 1–4) may be indicative of rapid population growth (Avise, 2000). Also, the star-like structures of the two most common haplotypes may be related to recent population expansion (see Feis et al., 2015). Interestingly, pennellids from the same individual host were sometimes more closely related to those from other hosts (even if collected in different years) than to each other. This could indicate that individuals within an infrapopulation have colonized their hosts during different events, separated in time and potentially in space, and belong to different genetic pools. The less defined structure of the phylogenetic trees, which display a comb shape, is also compatible with recent (and potentially ongoing) genetic exchange between populations, as supported by the structure of the reticulate and haplotype networks.

In all haplogroups, we found *Pennella* from both geographic regions (except for the exclusively north Pacific Haplogroup 1), levels of host dispersal (i.e. with or without interoceanic connectivity), and from multiple host species. Genetic variation was lower between geographic regions and host types (both dispersal level and taxon) than within each group. Firstly, the lack of differentiation by geographic region or degree of host dispersal could indicate high rates of genetic exchange across oceans (see below). Secondly, the extremely low host specificity of *Pennella* may explain why patterns of genetic diversity did not match host taxonomy; e.g. even the most closely related fish parasites were found on relatively distant taxa (i.e. scombriforms, beloniforms, and acropomatiforms; Near et al., 2012; Malmstrøm et al., 2016; Smith et al., 2022). The degree of host–parasite taxonomic congruence may increase with host specificity, and in parasites with direct life cycles, low dispersal, and limited (or no) free-living stages (Hafner et al., 2003; Nieberding et al., 2004). The opposite scenario seems to hold true for *Pennella* – i.e. a generalist parasite of marine vertebrates with a complex life cycle with free-living stages and some highly vagile hosts–, thus it seemingly benefits from great opportunities for dispersal. Note, however, that mating occurs at the intermediate host, where host specificity could be different.

Even if unattributable to geographic origin or host, genetic variation was significant between the five haplogroups (see above). Therefore, it seems unlikely that *Pennella* represents a strict panmictic population. Instead, gravid females of *Pennella* seem to show low specificity for definitive hosts and disperse across oceans with some definitive hosts. At a regional scale, earlier infective stages could exhibit greater specificity for intermediate hosts – where mating (genetic exchange) occurs, hence resulting in some degree of reproductive isolation. Interoceanic connectivity is likely facilitated by the dispersal of highly vagile definitive hosts, including the humpback whale (*Megaptera novaeangliae*), sei whale (*Balaenoptera borealis*), swordfish (*Xiphias gladius*), and ocean sunfish (*Mola mola*) (Table S2). In fact, it has been suggested that ocean sunfish are responsible for the range expansion of its helminth parasites into the Mediterranean Sea (Santoro et al., 2020). Host dispersal may contribute to the effect of the ‘high mixing in aquatic habitats’ hypothesis, which proposes that aquatic parasites with several hosts have multiple opportunities for mixing of unrelated individuals during transmission to the definitive host (Criscione and Blouin, 2006; Criscione et al., 2011). Moreover, the effect of host-mediated dispersal on the parasite’s genetic structure (e.g. Hedgecock et al., 2007; Fraija-Fernández et al., 2017) could be enhanced in species with high fecundity like the pennellids (e.g. Whitfield et al., 1988; Yumura et al., 2022), in which a relatively small proportion of adults may account for the bulk of reproduction of a population at a specific spatial and temporal scale (i.e. sweepstakes events, which are sometimes hard to detect; Hedgecock et al., 2007). Therefore, immigrants could release a great number of larvae into the new geographic region and lead to population expansion from a few animals, which is consistent with the aforementioned star shapes in the haplotype network (Nieberding et al., 2004). Other dispersal mechanisms for *Pennella* (e.g. independent swimming or drifting, or transport in ballast water; see Pagenkopp Lohan et al., 2022) seem unlikely, given the brief naupliar stage and presumably low vagility of the putative intermediate flatfish or cephalopod hosts (e.g. Arroyo et al., 2002; Izawa, 2019). Nonetheless, the identity and degree of dispersal of the intermediate hosts and the duration of the second infective stage (i.e. the inseminated adult female) remain unknown, hence we cannot rule out the additional effect of these stages on parasite dispersal.

In the future, genomic data or microsatellite markers could allow for kinship analyses, potentially overcoming the limitations of indirect methods such as *F*-statistics for investigating gene flow at diverse scales (e.g. Iacchei et al., 2013; Carroll et al., 2019). Even if COI has been applied successfully in analyses of cryptic species complexes (Hebert et al., 2004), and successfully among siphonostomatids, multilocus approaches provide a better understanding of cryptic structure (Criscione et al., 2011) and assist in the investigation of host–parasite phylogenetic congruence (e.g. Sweet et al., 2018). Lastly, gathering samples from other geographic locations could also allow for reconstructing gene flow via interpolation (Iacchei et al., 2013).

## Supporting information

Ten et al. supplementary material Ten et al. supplementary material

## Data Availability

New COI sequences have been uploaded to GenBank under Accession Numbers PP908425–PP908447.

## Supplementary material

The supplementary material for this article can be found at https://doi.org/10.1017/S0031182025000101.

## References

[ref1] Abaunza P, Arroyo NL and Preciado I (2001) A contribution to the knowledge on the morphometry and the anatomical characters of *Pennella balaenopterae* (Copepoda, Siphonostomatoida, Pennellidae), with special reference to the buccal complex. *Crustaceana* 74, 193–210.

[ref2] Anstensrud M (1992) Mate guarding and mate choice in two copepods, lernaeocera branchialis (L.)(Pennellidae) and *Lepeophtheirus pectoralis* (Müller)(Caligidae), parasitic on flounder. *Journal of Crustacean Biology* 12(1), 31–40.

[ref3] Arroyo NL, Abaunza P and Preciado I (2002) The first naupliar stage of *Pennella balaenopterae* Koren and Danielssen, 1877 (Copepoda: Siphonostomatoida, Pennellidae). *Sarsia* 87, 333–337.

[ref4] Avise JC (2000) *Phylogeography: the History and Formation of Species*: Cambridge, Massachusetts, United States: Harvard University Press.

[ref5] Bandelt HJ, Forster P and Rohl A (1999) Median-joining networks for inferring intraspecific phylogenies. *Molecular Biology and Evolution* 16(1), 37–48. 10331250 10.1093/oxfordjournals.molbev.a026036

[ref6] Blair C and Ané C (2020) Phylogenetic trees and networks can serve as powerful and complementary approaches for analysis of genomic data. *Systematic Biology* 69(3), 593–601. 31432090 10.1093/sysbio/syz056

[ref7] Boulding EG, DeWaard JR, Ang KP and Hebert PN (2009) Population genetic structure of the salmon louse, *lepeophtheirus salmonis* (krøyer) on wild and farmed salmonids around the Pacific coast of Canada. *Aquaculture Research* 40(8), 973–979.

[ref8] Boxshall G, Lester R, Grygier MJ, Hoeg JT, Glenner H, Shields JD, and Lützen J (2005) Crustacean parasites. In Rohde K (ed), *Marine parasitolog*. Collingwood, Australia: CSIRO Publishing, pp. 123-169.

[ref9] Bryant D and Huson DH (2023) NeighborNet: Improved algorithms and implementation. *Frontiers in Bioinformatics* 3, 1178600. 37799982 10.3389/fbinf.2023.1178600 PMC10548196

[ref10] Carroll EL, Alderman R, Bannister JL, Bérube M, Best PB, Boren L, Baker CS, Constantine R, Findlay K, Harcourt R, Lemaire L, Palsbøll PJ, Patenaude NJ, Rowntree VJ, Seger J, Steel D, Valenzuela LO, Watson M and Gaggiotti OE (2019) Incorporating non-equilibrium dynamics into demographic history inferences of a migratory marine species. *Heredity* 122(1), 53–68. 29720718 10.1038/s41437-018-0077-y PMC6288115

[ref11] Castro-Romero R, Montes MM, Martorelli SR, Sepulveda D, Tapia S and Martínez-Aquino A (2016) Integrative taxonomy of *peniculus, metapeniculus*, and *Trifur* (siphonostomatoida: Pennellidae), copepod parasites of marine fishes from chile: Species delimitation analyses using DNA barcoding and morphological evidence. *Systematics and Biodiversity* 14(5), 466–483.

[ref12] Chaieb O, Ten S and Aznar FJ (2024) *Pennella balaenoptera* actively select injured cetacean skin as attachment sites, making them potentially useful forensic tags. *Diseases of Aquatic Organisms* 158, 195–200. 38934259 10.3354/dao03796

[ref13] Clement M, Posada DCKA and Crandall KA (2000) TCS: A computer program to estimate gene genealogies. *Molecular Ecology* 9(10), 1657–1659. 11050560 10.1046/j.1365-294x.2000.01020.x

[ref14] Clement M, Snell Q, Walke P, Posada D and Crandall K (2002) TCS: Estimating gene genealogies. Proceeding 16th International Parallel Distributed Processing Symposium, 184.

[ref15] Criscione CD and Blouin MS (2006) Minimal selfing, few clones, and no among‐host genetic structure in a hermaphroditic parasite with asexual larval propagation. *Evolution* 60(3), 553–562. 16637500

[ref16] Criscione CD, Vilas R, Paniagua E and Blouin MS (2011) More than meets the eye: Detecting cryptic microgeographic population structure in a parasite with a complex life cycle. *Molecular Ecology* 20(12), 2510–2524. 21535278 10.1111/j.1365-294X.2011.05113.x

[ref17] Dailey MD, Haulena M and Lawrence J (2002) First report of a parasitic copepod (*Pennella balaenopterae*) infestationin a pinniped. *Journal of Zoo and Wildlife Medicine* 33(1), 62–65. 12216795 10.1638/1042-7260(2002)033[0062:FROAPC]2.0.CO;2

[ref18] Darriba D, Taboada GL, Doallo R and Posada D (2012) jModelTest 2: More models, new heuristics and parallel computing. *Nature Methods* 9(8), 772. 10.1038/nmeth.2109 PMC4594756 22847109

[ref19] Dippenaar SM (2009) Estimated molecular phylogenetic relationships of six siphonostomatoid families (Copepoda) symbiotic on elasmobranchs. *Crustaceana* 82(12), 1547.

[ref20] Dippenaar SM, Mathibela RB and Bloomer P (2010) Cytochrome oxidase I sequences reveal possible cryptic diversity in the cosmopolitan symbiotic copepod *nesippus orientalis* heller, 1868 (pandaridae: Siphonostomatoida) on elasmobranch hosts from the kwazulu-natal coast of South Africa. *Experimental Parasitology* 125(1), 42–50. 19723521 10.1016/j.exppara.2009.08.017

[ref21] Dumas P, Barbut J, Le Ru B, Silvain JF, Clamens AL, d’Alençon E and Kergoat GJ (2015) Phylogenetic molecular species delimitations unravel potential new species in the pest genus *spodoptera* guenée, 1852 (lepidoptera, noctuidae). *PLoS One* 10(4), e0122407. 25853412 10.1371/journal.pone.0122407 PMC4390195

[ref22] Excoffier L and Lischer HE (2010) Arlequin suite ver 3.5: A new series of programs to perform population genetics analyses under Linux and Windows. *Molecular Ecology Resources* 10(3), 564–567. 21565059 10.1111/j.1755-0998.2010.02847.x

[ref23] Feis ME, Thieltges DW, Olsen JL, de Montaudouin X, Jensen KT, Bazaïri H, Culloty S and Luttikhuizen PC (2015) The most vagile host as the main determinant of population connectivity in marine macroparasites. *Marine Ecology Progress Series* 520, 85–99.

[ref24] Fraija-Fernández N, Fernández M, Lehnert K, Raga JA, Siebert U and Aznar FJ (2017) Long-distance travellers: Phylogeography of a generalist parasite, *pholeter gastrophilus*, from cetaceans. *Plos One* 12(1), e0170184. 28085945 10.1371/journal.pone.0170184 PMC5234839

[ref25] Fraija-Fernández N, Hernández-Hortelano A, Ahuir-Baraja AE, Raga JA and Aznar FJ (2018) Taxonomic status and epidemiology of the mesoparasitic copepod *Pennella balaenoptera* in cetaceans from the western mediterranean. *Diseases of Aquatic Organisms* 128, 249–258. 29862982 10.3354/dao03226

[ref26] Guindon S and Gascuel O (2003) A simple, fast and accurate method to estimate large phylogenies by maximum-likelihood. *Systematic Biology* 52, 696–704. 14530136 10.1080/10635150390235520

[ref27] Hafner MS, Demastes JW, Spradling TA, Reed DL, and Page RDM (2003) *Cophylogeny between Pocket Gophers and Chewing Lice. Tangled Trees: phylogeny, Cospeciation and Coevolution*: Chicago, Illinois, U.S: Chicago University Chicago Press. 195–220.

[ref28] Hebert PD, Penton EH, Burns JM, Janzen DH and Hallwachs W (2004) Ten species in one: DNA barcoding reveals cryptic species in the neotropical skipper butterfly *Astraptes fulgerator*. *Proceedings of the National Academy of Sciences* 101(41), 14812–14817. 10.1073/pnas.0406166101 PMC522015 15465915

[ref29] Hedgecock D, Barber PH and Edmands S (2007) Genetic approaches to measuring connectivity. *Oceanography* 20, 70–79.

[ref30] Hogans WE (1987) Morphological variation in *Pennella balaenoptera* and *P. filosa* (Copepoda: Pennellidae) with a review of the genus *Pennella* Oken, 1816 parasitic on cetacea. *Bulletin of Marine Science* 40(3), 442–453.

[ref31] Hogans WE (2017) Review of *Pennella* Oken, 1816 (Copepoda: Pennellidae) with a description of *Pennella benzi* sp. nov., a parasite of escolar, *Lepidocybium flavobrunneum* (Pisces) in the northwest Atlantic Ocean. *Zootaxa* 4244, 1–38. 28610128 10.11646/zootaxa.4244.1.1

[ref32] Huelsenbeck JP, Ronquist F, and Hall B (2001) An introduction to bayesian inference of phylogeny. 1–7.

[ref33] Iacchei M, Ben‐Horin T, Selkoe KA, Bird CE, García‐Rodríguez FJ and Toonen RJ (2013) Combined analyses of kinship and FST suggest potential drivers of chaotic genetic patchiness in high gene‐flow populations. *Molecular Ecology* 22(13), 3476–3494. 23802550 10.1111/mec.12341 PMC3749441

[ref34] Izawa K (2019) Redescription of *Lernaeenicus ramosus* Kirtisinghe, 1956 (Copepoda, Siphonostomatoida, Pennellidae), with description of its male and the postnaupliar developmental stages. *Crustaceana* 92(1), 119–128.

[ref35] Kabata Z (1979) *Parasitic Copepoda of British Fishes*. The Ray Society: London.

[ref36] Leigh JW and Bryant D (2015) POPART: Full-feature software for haplotype network construction. *Methods in Ecology and Evolution* 6(9), 1110–1116.

[ref37] Lovy J and Friend SE (2020) Black sea bass are a host in the developmental cycle of *lernaeenicus radiatus* (Copepoda: Pennellidae): Insights into parasite morphology, gill pathology and genetics. *Parasitology* 147(4), 478–490. 31852554 10.1017/S0031182019001781 PMC10317654

[ref38] Malmstrøm M, Matschiner M, Tørresen OK, Star B, Snipen LG, Hansen TF, Baalsrud HT, Nederbragt AJ, Hanel R, Salzburger W, Stenseth NC, Jakobsen KS and Jentoft S (2016) Evolution of the immune system influences speciation rates in teleost fishes. *Nature Genetics.* 48(10), 1204–1210. 27548311 10.1038/ng.3645

[ref39] Mangena T, Jordaan BP and Dippenaar SM (2014) Phylogenetic relationships and genetic diversity of nemesis risso, 1826 species found on different elasmobranch host species off the kwazulu-natal coast, South Africa. *African Journal of Marine Science* 36(2), 163–173.

[ref40] Miller MA, Pfeiffer W and Schwartz T (2010) Creating the CIPRES science gateway for inference of large phylogenetic trees. In 2010 gateway computing environments workshop (GCE). Ieee, 1–8.

[ref41] Morales-Serna FN, Pinacho-Pinacho CD, Gómez S and de León GPP (2014) Diversity of sea lice (Copepoda: Caligidae) parasitic on marine fishes with commercial and aquaculture importance in chamela bay, Pacific coast of Mexico by using morphology and DNA barcoding, with description of a new species of *Caligus*. *Parasitology International* 63(1), 69–79. 24042060 10.1016/j.parint.2013.09.005

[ref42] Muñoz G, Landaeta MF, Palacios-Fuentes P, López Z and González MT (2015) Parasite richness in fish larvae from the nearshore waters of central and northern Chile. *Folia Parasitologica* 62, 1–12. 10.14411/fp.2015.029 26040465

[ref43] Near TJ, Eytan RI, Dornburg A, Kuhn KL, Moore JA, Davis MP, Wainwright PC, Friedman M and Smith WL (2012) Resolution of ray-finned fish phylogeny and timing of diversification. *Proceedings of the National Academy of Sciences* 109(34), 13698–13703. 10.1073/pnas.1206625109 PMC3427055 22869754

[ref44] Nieberding C, Morand S, Libois R and Michaux JR (2004) A parasite reveals cryptic phylogeographic history of its host. *Proceedings of the Royal Society of London Series B Biological Sciences* 271(1557), 2559–2568. 10.1098/rspb.2004.2930 PMC1691906 15615681

[ref45] Øines Ø and Heuch PA (2005) Identification of sea louse species of the genus *Caligus* using mtDNA. *Journal of the Marine Biological Association of the United Kingdom* 85, 73–79.

[ref46] Øines Ø and Schram T (2008) Intra- or inter-specific difference in genotypes of *caligus elongatus* nordmann 1832? *Acta Parasitologica* 53, 93–105.

[ref47] Pagenkopp Lohan KM, Darling JA and Ruiz GM (2022) International shipping as a potent vector for spreading marine parasites. *Diversity and Distributions* 28(9), 1922–1933. 38269301 10.1111/ddi.13592 PMC10807284

[ref48] Pons J, Barraclough TG, Gomez-Zurita J, Cardoso A, Duran DP, Hazell S, Kamoun S, Sumlin WD and Vogler AP (2006) Sequence-based species delimitation for the DNA taxonomy of undescribed insects. *Systematic Biology* 55(4), 595–609. 16967577 10.1080/10635150600852011

[ref49] Porter TM and Hajibabaei M (2021) Profile hidden Markov model sequence analysis can help remove putative pseudogenes from DNA barcoding and metabarcoding datasets. *BMC Bioinformatics* 22(1), 256. 34011275 10.1186/s12859-021-04180-x PMC8136176

[ref50] Puillandre N, Brouillet S and Achaz G (2021) ASAP: Assemble species by automatic partitioning. *Molecular Ecology Resources* 21(2), 609–620. 33058550 10.1111/1755-0998.13281

[ref51] Puillandre N, Lambert A, Brouillet S and Achaz GJME (2012) ABGD, automatic barcode gap discovery for primary species delimitation. *Molecular Ecology* 21(8), 1864–1877. 21883587 10.1111/j.1365-294X.2011.05239.x

[ref52] Rambaut A (2010) FigTree v1.3.1. Institute of Evolutionary Biology, University of Edinburgh, Edinburgh http://tree.bio.ed.ac.uk/software/figtree/ (accessed 14 March 2024).

[ref53] Rambaut A, and Drummond AJ (2009) Tracer v1.5, http://tree.bio.ed.ac.uk/software/tracer/ (accessed 23 March 2024).

[ref54] Santoro M, Palomba M, Mattiucci S, Osca D and Crocetta F (2020) New parasite records for the sunfish *Mola mola* in the Mediterranean sea and their potential use as biological tags for long-distance host migration. *Frontiers in Veterinary Science* 7, 579728. 33195589 10.3389/fvets.2020.579728 PMC7641614

[ref55] Skern-Mauritzen R, Torrissen O and Glover KA (2014) Pacific and Atlantic *Lepeophtheirus salmonis* (Krøyer, 1838) are allopatric subspecies: *Lepeophtheirus salmonis salmonis* and *L. salmonis oncorhynchi* subspecies novo. *BMC Genetics* 15, 1–9. 24628716 10.1186/1471-2156-15-32 PMC4007600

[ref56] Smith WL, Ghedotti MJ, Domínguez-Domínguez O, McMahan CD, Espinoza E, Martin RP, Girard MG and Davis MP (2022) Investigations into the ancestry of the grape-eye seabass (*Hemilutjanus macrophthalmos*) reveal novel limits and relationships for the acropomatiformes (Teleostei: Percomorpha). *Neotropical Ichthyology* 20, e210160.

[ref57] Stamatakis A (2014) RAxML version 8: A tool for phylogenetic analysis and post-analysis of large phylogenies. *Bioinformatics* 30(9), 1312–1313. 24451623 10.1093/bioinformatics/btu033 PMC3998144

[ref58] Suyama S, Kakehi S, Yanagimoto T and Chow S (2020) Infection of the pacific saury *Cololabis saira* (brevoort, 1856) (Teleostei: Beloniformes: Scomberesocidae) by *Pennella* sp. (Copepoda: Siphonostomatoida: Pennellidae) south of the subarctic front. *The Journal of Crustacean Biology* 40(4), 384–389.

[ref59] Suyama S, Masuda Y, Yanagimoto T and Chow S (2019) Genetic and morphological variation in *Pennella* sp. (Copepoda: Siphonostomatoida) collected from Pacific saury. *Cololabis Saira. Marine Biodiversity* 49(3), 1233–1245.

[ref60] Suyama S, Miyamoto H and Fuji T (2021a) Infection by the parasitic copepod *Pennella* sp. induces mortality in the Pacific saury *Cololabis saira*. *Fisheries Science* 87(2), 187–202.

[ref61] Suyama S, Yanagimoto T, Nakai K, Tamura T, Shiozaki K, Ohshimo S and Chow S (2021b) A taxonomic revision of *Pennella* Oken, 1815 based on morphology and genetics (Copepoda: Siphonostomatoida: Pennellidae). *Journal of Crustacean Biology* 41, ruab040.

[ref62] Sweet AD, Bush SE, Gustafsson DR, Allen JM, DiBlasi E, Skeen HR, Weckstein JD and Johnson KP (2018) Host and parasite morphology influence congruence between host and parasite phylogenies. *International Journal for Parasitology* 48(8), 641–648. 29577890 10.1016/j.ijpara.2018.01.007

[ref63] Tang CQ, Humphreys AM, Fontaneto D and Barraclough TG (2014) Effects of phylogenetic reconstruction method on the robustness of species delimitation using single‐locus data. *Methods in Ecology and Evolution* 5(10), 1086–1094. 25821577 10.1111/2041-210X.12246 PMC4374709

[ref64] Ten S, Raga JA and Aznar FJ (2022) Epibiotic fauna on cetaceans worldwide: A systematic review of records and indicator potential. *Frontiers in Marine Science* 9, 846558.

[ref65] Turner W (1905) On *Pennella balaenopterae*: A crustacean, parasitic on a finner whale, *Balaenoptera musculus*. *Transactions of the Royal Society of Edinburgh* 2, 409–434.

[ref66] Vecchione A and Aznar FJ (2014) The mesoparasitic copepod *Pennella balaenopterae* and its significance as a visible indicator of health status in dolphins (Delphinidae): A review. *Journal of Marine Animal Ecology* 7, 4–11.

[ref67] Whitfield PJ, Pilcher MW, Grant HJ and Riley J (1988) Experimental studies on the development of *Lernaeocera branchialis* (Copepoda: Pennellidae): Population processes from egg production to maturation on the flatfish host. In Boxshall GA and Schminke HK (eds), Biology of Copepods: Proceedings of the Third International Conference on Copepoda. Springer Netherlands, 579–586.

[ref68] Xue L, Moreira JD, Smith KK and Fetterman JL (2023) The mighty NUMT: Mitochondrial DNA flexing its code in the nuclear genome. *Biomolecules* 13(5), 753. 37238623 10.3390/biom13050753 PMC10216076

[ref69] Yumura N, Adachi K, Nitta M, Kondo Y, Komeda S, Wakabayashi K, Fukuchi J, Boxshall GA and Ohtsuka S (2022) Exploring evolutionary trends within the *Pennellidae* (Copepoda: Siphonostomatoida) using molecular data. *Systematic Parasitology* 99(4), 477–489. 35583766 10.1007/s11230-022-10040-w

[ref70] Zhang J, Kapli P, Pavlidis P and Stamatakis A (2013) A general species delimitation method with applications to phylogenetic placements. *Bioinformatics* 29, 2869–2876. 23990417 10.1093/bioinformatics/btt499 PMC3810850

