# Supplementary material for: Phylogeography of Pennella (Copepoda: Siphonostomatoida: Pennellidae) indicates interoceanic dispersal mediated by cetacean and fish hosts
Source: Parasitology. 2025 Jan 28;152(2):195–204. doi: 10.1017/S0031182025000101 (PMC12089445; doi:10.1017/S0031182025000101)
Supplement: Ten et al. supplementary material [file S0031182025000101sup001.docx]

Phylogeography of *Pennella* (Copepoda: Siphonostomatoida: Pennellidae) indicates interoceanic dispersal mediated by cetacean and fish hosts

Sofía Ten, Rachel Vanessa Pool, Juan Antonio Raga, Andrew D. Sweet, Francisco Javier Aznar Avendaño

# Supplementary Material

## Supplementary Figures

**Figure S1.** Maximum Likelihood (ML) tree based on COI sequences of the genus *Pennella* from the North Pacific and western Mediterranean. Labels indicate ML bootstrap support values, with 1M replicates; values <70% are not shown.. Color bars indicate the clusters of haplotypes depicted in the haplotype network on the left (Fig. 3). See accession numbers and sequence details in Fig. 2 and Table S1.

**Figure S2.** Reticulate relationships among *Pennella* spp. COI sequences based on K2P distances. Five major clusters were identified; see the main text for details.

**Figure S3.** Parsimony haplotype network of COI sequences from the marine parasitic copepod *Pennella*. Haplotype frequency is proportional to the circle area. Colors represent the host taxon harboring each of the samples, including teleost orders and cetacean superfamilies. An enlarged vision of cluster I is displayed on the right; see the main text for details about the clusters.

## Supplementary Tables

**Table S1.** COI gene haplotypes (H) of *Pennella* specimens from the western Mediterranean (WM) and North Pacific (NP). The collection year, when unknown, was inferred from the available information on each reference; [1] Suyama et al., 2021b; [2] This study, [3] Fraija-Fernández et al., 2018. Classification by ‘groups’ based on morphology and phylogenetic criteria *sensu* [1], whereas ‘clusters’ were identified on a haplotype network in [2]. For each specimen, the GenBank accession number, morphological identification, and host identity are provided; host individuals are identified whenever possible. Identification of *Pennella* sp. 1 sensu [1]; *P. filosa** refers to the synonym *P. balaenoptera*.

| **H** | **Group [1] / Cluster [2]** | **Accession number** | **Morphological identification** | **Region** | **Host**  **taxon** | **Host species^§^** | **Collection year** | **Ref.** |
| --- | --- | --- | --- | --- | --- | --- | --- | --- |
| I | I / 1 | LC638573 | Pennella sp. 1 | NP | Tetraodontiformes | Mm5 | 2012-2020 | [1] |
| I |  | LC638580 | Pennella sp. 1 | NP | Acropomatiformes | Pw1 | 2012-2020 | [1] |
| I |  | LC638581 | Pennella sp. 1 | NP | Acropomatiformes | Pw2 | 2012-2020 | [1] |
| I |  | LC638585 | *Pennella* sp. | NP | Beloniformes | Cs1 | 2012-2020 | [1] |
| I |  | LC638615 | *Pennella* sp. | NP | Beloniformes | Cs13 | 2012-2020 | [1] |
| I |  | LC638618 | *Pennella* sp. | NP | Beloniformes | Cs15 | 2012-2020 | [1] |
| I |  | LC638619 | *Pennella* sp. | NP | Beloniformes | Cs15 | 2012-2020 | [1] |
| I |  | LC638622 | Pennella sp. 1 | NP | Carangiformes | Sq1 | 2012-2020 | [1] |
| II |  | LC638574 | Pennella sp. 1 | NP | Tetraodontiformes | Mm5 | 2012-2020 | [1] |
| III |  | LC638575 | Pennella sp. 1 | NP | Tetraodontiformes | Mm* | 2012-2020 | [1] |
| III |  | LC638577 | Pennella sp. 1 | NP | Tetraodontiformes | Mm* | 2012-2020 | [1] |
| IV |  | LC638576 | Pennella sp. 1 | NP | Tetraodontiformes | Mm* | 2012-2020 | [1] |
| V |  | LC638578 | Pennella sp. 1 | NP | Tetraodontiformes | Mm* | 2012-2020 | [1] |
| VI |  | LC638579 | Pennella sp. 1 | NP | Tetraodontiformes | Mm* | 2012-2020 | [1] |
| VII |  | LC638582 | Pennella sp. 1 | NP | Acropomatiformes | Pw3 | 2012-2020 | [1] |
| VIII |  | LC638583 | Pennella sp. 1 | NP | Acropomatiformes | Pw4 | 2012-2020 | [1] |
| IX |  | LC638584 | *Pennella* sp. | NP | Beloniformes | Cs1 | 2012-2020 | [1] |
| X |  | LC638586 | *Pennella* sp. | NP | Beloniformes | Cs2 | 2012-2020 | [1] |
| XI |  | LC638587 | *Pennella* sp. | NP | Beloniformes | Cs2 | 2012-2020 | [1] |
| XII |  | LC638588 | *Pennella* sp. | NP | Beloniformes | Cs2 | 2012-2020 | [1] |
| XIII |  | LC638589 | *Pennella* sp. | NP | Beloniformes | Cs2 | 2012-2020 | [1] |
| XIV |  | LC638590 | *Pennella* sp. | NP | Beloniformes | Cs3 | 2012-2020 | [1] |
| XV |  | LC638591 | *Pennella* sp. | NP | Beloniformes | Cs3 | 2012-2020 | [1] |
| XVI |  | LC638592 | *Pennella* sp. | NP | Beloniformes | Cs4 | 2012-2020 | [1] |
| XVI |  | LC638593 | *Pennella* sp. | NP | Beloniformes | Cs4 | 2012-2020 | [1] |
| XVII |  | LC638594 | *Pennella* sp. | NP | Beloniformes | Cs5 | 2012-2020 | [1] |
| XVIII |  | LC638595 | *Pennella* sp. | NP | Beloniformes | Cs5 | 2012-2020 | [1] |
| XIX |  | LC638596 | *Pennella* sp. | NP | Beloniformes | Cs5 | 2012-2020 | [1] |
| XX |  | LC638597 | *Pennella* sp. | NP | Beloniformes | Cs5 | 2012-2020 | [1] |
| XXI |  | LC638598 | *Pennella* sp. | NP | Beloniformes | Cs5 | 2012-2020 | [1] |
| XXII |  | LC638599 | *Pennella* sp. | NP | Beloniformes | Cs5 | 2012-2020 | [1] |
| XXIII |  | LC638600 | *Pennella* sp. | NP | Beloniformes | Cs6 | 2012-2020 | [1] |
| XXIV |  | LC638601 | *Pennella* sp. | NP | Beloniformes | Cs7 | 2012-2020 | [1] |
| XXV |  | LC638602 | *Pennella* sp. | NP | Beloniformes | Cs7 | 2012-2020 | [1] |
| XXVI |  | LC638603 | *Pennella* sp. | NP | Beloniformes | Cs7 | 2012-2020 | [1] |
| XXVII |  | LC638604 | *Pennella* sp. | NP | Beloniformes | Cs8 | 2012-2020 | [1] |
| XXVIII |  | LC638605 | *Pennella* sp. | NP | Beloniformes | Cs8 | 2012-2020 | [1] |
| XXVIII |  | LC638606 | *Pennella* sp. | NP | Beloniformes | Cs8 | 2012-2020 | [1] |
| XXIX |  | LC638607 | *Pennella* sp. | NP | Beloniformes | Cs9 | 2012-2020 | [1] |
| XXIX |  | LC638608 | *Pennella* sp. | NP | Beloniformes | Cs9 | 2012-2020 | [1] |
| XXX |  | LC638609 | *Pennella* sp. | NP | Beloniformes | Cs10 | 2012-2020 | [1] |
| XXXI |  | LC638610 | *Pennella* sp. | NP | Beloniformes | Cs10 | 2012-2020 | [1] |
| XXXII |  | LC638611 | *Pennella* sp. | NP | Beloniformes | Cs11 | 2012-2020 | [1] |
| XXXIII |  | LC638612 | *Pennella* sp. | NP | Beloniformes | Cs12 | 2012-2020 | [1] |
| XXXIV |  | LC638613 | *Pennella* sp. | NP | Beloniformes | Cs12 | 2012-2020 | [1] |
| XXXV |  | LC638614 | *Pennella* sp. | NP | Beloniformes | Cs12 | 2012-2020 | [1] |
| XXXVI |  | LC638616 | *Pennella* sp. | NP | Beloniformes | Cs14 | 2012-2020 | [1] |
| XXXVI |  | LC638617 | *Pennella* sp. | NP | Beloniformes | Cs14 | 2012-2020 | [1] |
| XXXVII |  | LC638620 | Pennella sp. 1 | NP | Carangiformes | Sq1 | 2012-2020 | [1] |
| XXXVIII |  | LC638621 | Pennella sp. 1 | NP | Carangiformes | Sq1 | 2012-2020 | [1] |
| XXXIX | II / 2 | PP908436 | *P. filosa** | WM | Mysticeti | Bp4 | 1980 | [2] |
| XL |  | PP908428 | *P. filosa** | WM | Mysticeti | Bp1 | 2011 | [2] |
| XLI |  | PP908443 | *P. filosa** | WM | Mysticeti | Bp2 | 2020 | [2] |
| XLI |  | PP908432 | *P. filosa** | WM | Mysticeti | Bp1 | 2011 | [2] |
| XLI |  | PP908434 | *P. filosa** | WM | Mysticeti | Bp1 | 2011 | [2] |
| XLII |  | PP908439 | *P. filosa** | WM | Mysticeti | Bp2 | 2020 | [2] |
| XLIII |  | PP908440 | *P. filosa** | WM | Mysticeti | Bp2 | 2020 | [2] |
| XLIV |  | PP908441 | *P. filosa** | WM | Mysticeti | Bp2 | 2020 | [2] |
| XLV |  | PP908445 | *P. filosa** | WM | Mysticeti | Bp3 | 2021 | [2] |
| XLVI |  | PP908446 | *P. filosa** | WM | Mysticeti | Bp3 | 2021 | [2] |
| XLVI |  | LC642595 | *P. filosa* | NP | Scombriformes | Lf2 | 2012-2020 | [1] |
| XLVII |  | PP908444 | *P. filosa** | WM | Mysticeti | Mn2 | 2019 | [2] |
| XLVII |  | PP908426 | *P. filosa** | WM | Mysticeti | Mn2 | 2019 | [2] |
| XLVII |  | PP908430 | *P. filosa** | WM | Mysticeti | Mn2 | 2019 | [2] |
| XLVIII |  | PP908425 | *P. filosa** | WM | Mysticeti | Mn1 | 2022 | [2] |
| XLIX |  | PP908427 | *P. filosa** | WM | Mysticeti | Mn1 | 2022 | [2] |
| L |  | PP908429 | *P. filosa** | WM | Mysticeti | Mn1 | 2022 | [2] |
| LI |  | PP908433 | *P. filosa** | WM | Mysticeti | Mn1 | 2022 | [2] |
| LII |  | PP908435 | *P. filosa** | WM | Mysticeti | Mn1 | 2022 | [2] |
| LII |  | LC642568 | *P.balaenoptera* | NP | Mysticeti | Be1 | 2012-2020 | [1] |
| LII |  | LC642569 | *P.balaenoptera* | NP | Mysticeti | Be1 | 2012-2020 | [1] |
| LII |  | LC642571 | *Pennella* sp. | NP | Mysticeti | Bb2 | 2012-2020 | [1] |
| LII |  | LC642573 | *Pennella* sp. | NP | Mysticeti | Bb2 | 2012-2020 | [1] |
| LII |  | LC642574 | *P.balaenoptera* | NP | Mysticeti | Be2 | 2012-2020 | [1] |
| LII |  | LC642576 | *P.balaenoptera* | NP | Mysticeti | Be2 | 2012-2020 | [1] |
| LII |  | LC642578 | *P.balaenoptera* | NP | Mysticeti | Be2 | 2012-2020 | [1] |
| LII |  | LC642588 | *P. filosa* | NP | Carangiformes | Ka2 | 2012-2020 | [1] |
| LII |  | LC642590 | *P. benzi* | NP | Scombriformes | Lf1 | 2012-2020 | [1] |
| LII |  | LC642602 | *Pennella* sp. | NP | Tetraodontiformes | Mm4 | 2012-2020 | [1] |
| LII |  | LC642616 | *P. filosa* | NP | Tetraodontiformes | Tr3 | 2012-2020 | [1] |
| LII |  | LC642618 | *P. filosa* | NP | Tetraodontiformes | Tr3 | 2012-2020 | [1] |
| LIII |  | PP908437 | *P. filosa** | WM | Mysticeti | Mn1 | 2022 | [2] |
| LIV |  | PP908438 | *P. filosa** | WM | Mysticeti | Mn1 | 2022 | [2] |
| LV |  | MG701282 | *P. filosa* | WM | Carangiformes | Xg4 | 2016 | [3] |
| LVI |  | MG701285 | *P. filosa* | WM | Carangiformes | Xg4 | 2016 | [3] |
| LVII |  | MG701286 | *P. filosa* | WM | Carangiformes | Xg4 | 2016 | [3] |
| LVIII |  | LC642610 | *P. filosa* | NP | Tetraodontiformes | Mm5 | 2012-2020 | [1] |
| LVIII |  | MG701287 | *P. filosa** | WM | Odontoceti | Dd | 2012-2020 | [3] |
| LVIII |  | MG701289 | *P. filosa** | WM | Odontoceti | Gg1 | 2012-2020 | [3] |
| LVIII |  | MG701290 | *P. filosa** | WM | Odontoceti | Dd | 2012-2020 | [3] |
| LVIII |  | MG701291 | *P. filosa** | WM | Odontoceti | Dd | 2012-2020 | [3] |
| LVIII |  | MG701292 | *P. filosa** | WM | Odontoceti | Gm1 | 2012-2020 | [3] |
| LIX |  | LC642570 | *Pennella* sp. | NP | Mysticeti | Bb1 | 2012-2020 | [1] |
| LX |  | LC642572 | *Pennella* sp. | NP | Mysticeti | Bb2 | 2012-2020 | [1] |
| LXI |  | LC642575 | *P.balaenoptera* | NP | Mysticeti | Be2 | 2012-2020 | [1] |
| LXII |  | LC642577 | *P.balaenoptera* | NP | Mysticeti | Be2 | 2012-2020 | [1] |
| LXII |  | LC642617 | *P. filosa* | NP | Tetraodontiformes | Tr3 | 2012-2020 | [1] |
| LXIII |  | LC642579 | *P. filosa* | NP | Carangiformes | Ch1 | 2012-2020 | [1] |
| LXIII |  | LC642580 | *P. filosa* | NP | Carangiformes | Ch1 | 2012-2020 | [1] |
| LXIII |  | LC642615 | *P. filosa* | NP | Tetraodontiformes | Tr2 | 2012-2020 | [1] |
| LXIV |  | LC642581 | *P. filosa* | NP | Carangiformes | Ka1 | 2012-2020 | [1] |
| LXV |  | LC642582 | *P. filosa* | NP | Carangiformes | Ka1 | 2012-2020 | [1] |
| LXVI |  | LC642583 | *P. filosa* | NP | Carangiformes | Ka1 | 2012-2020 | [1] |
| LXVII |  | LC642584 | *P. filosa* | NP | Carangiformes | Ka1 | 2012-2020 | [1] |
| LXVIII |  | LC642585 | *P. filosa* | NP | Carangiformes | Ka1 | 2012-2020 | [1] |
| LXIX |  | LC642586 | *P. filosa* | NP | Carangiformes | Ka1 | 2012-2020 | [1] |
| LXX |  | LC642587 | *P. filosa* | NP | Carangiformes | Ka1 | 2012-2020 | [1] |
| LXXI |  | LC642589 | *P. benzi* | NP | Scombriformes | Lf1 | 2012-2020 | [1] |
| LXXII |  | LC642591 | *P. benzi* | NP | Scombriformes | Lf1 | 2012-2020 | [1] |
| LXXIII |  | LC642592 | *P. filosa* | NP | Scombriformes | Lf2 | 2012-2020 | [1] |
| LXXIV |  | LC642593 | *P. filosa* | NP | Scombriformes | Lf2 | 2012-2020 | [1] |
| LXXV |  | LC642594 | *P. filosa* | NP | Scombriformes | Lf2 | 2012-2020 | [1] |
| LXXVI |  | LC642596 | *P. filosa* | NP | Scombriformes | Lf2 | 2012-2020 | [1] |
| LXXVII |  | LC642597 | *P. filosa* | NP | Scombriformes | Lf2 | 2012-2020 | [1] |
| LXXVIII |  | LC642598 | *P. filosa* | NP | Tetraodontiformes | Mm2 | 2012-2020 | [1] |
| LXXIX |  | LC642599 | *P. filosa* | NP | Tetraodontiformes | Mm3 | 2012-2020 | [1] |
| LXXIX |  | LC642601 | *P. filosa* | NP | Tetraodontiformes | Mm3 | 2012-2020 | [1] |
| LXXX |  | LC642600 | *P. filosa* | NP | Tetraodontiformes | Mm3 | 2012-2020 | [1] |
| LXXXI |  | LC642603 | *Pennella* sp. | NP | Tetraodontiformes | Mm4 | 2012-2020 | [1] |
| LXXXII |  | LC642604 | *Pennella* sp. | NP | Tetraodontiformes | Mm4 | 2012-2020 | [1] |
| LXXXIII |  | LC642605 | *Pennella* sp. | NP | Tetraodontiformes | Mm4 | 2012-2020 | [1] |
| LXXXIV |  | LC642606 | *Pennella* sp. | NP | Tetraodontiformes | Mm4 | 2012-2020 | [1] |
| LXXXV |  | LC642607 | *Pennella* sp. | NP | Tetraodontiformes | Mm4 | 2012-2020 | [1] |
| LXXXVI |  | LC642608 | *Pennella* sp. | NP | Tetraodontiformes | Mm4 | 2012-2020 | [1] |
| LXXXVII |  | LC642609 | *Pennella* sp. | NP | Tetraodontiformes | Mm4 | 2012-2020 | [1] |
| LXXXVIII |  | LC642611 | *P. filosa* | NP | Tetraodontiformes | Mm* | 2012-2020 | [1] |
| LXXXIX |  | LC642612 | *P. filosa* | NP | Tetraodontiformes | Mm* | 2012-2020 | [1] |
| XC |  | LC642613 | *P. filosa* | NP | Tetraodontiformes | Tr1 | 2012-2020 | [1] |
| XCI |  | LC642614 | *P. filosa* | NP | Tetraodontiformes | Tr2 | 2012-2020 | [1] |
| XCII |  | LC642619 | *P. filosa* | NP | Tetraodontiformes | Tr3 | 2012-2020 | [1] |
| XCIII |  | LC642620 | *P. filosa* | NP | Tetraodontiformes | Tr3 | 2012-2020 | [1] |
| XCIV |  | LC642621 | *P. filosa* | NP | Tetraodontiformes | Tr3 | 2012-2020 | [1] |
| XCV |  | LC642622 | *P. filosa* | NP | Tetraodontiformes | Tr4 | 2012-2020 | [1] |
| XCVI |  | LC642623 | *P. instructa* | NP | Carangiformes | Xg1 | 2012-2020 | [1] |
| XCVII |  | LC642624 | *P. instructa* | NP | Carangiformes | Xg1 | 2012-2020 | [1] |
| XCVIII |  | LC642625 | *P. instructa* | NP | Carangiformes | Xg1 | 2012-2020 | [1] |
| XCIX |  | LC642626 | *P. instructa* | NP | Carangiformes | Xg1 | 2012-2020 | [1] |
| C |  | LC642627 | *P. filosa* | NP | Carangiformes | Xg2 | 2012-2020 | [1] |
| CI |  | LC642628 | *P. filosa* | NP | Carangiformes | Xg2 | 2012-2020 | [1] |
| CII | III / 3 | PP908431 | *P. filosa** | WM | Mysticeti | Mn1 | 2022 | [2] |
| CIII |  | MZ934350 | *Pennella* sp. | NP | Mysticeti | Bb1 | 2012-2020 | [1] |
| CIV |  | MZ934351 | *Pennella* sp. | NP | Mysticeti | Bb1 | 2012-2020 | [1] |
| CV |  | MZ934352 | *P. filosa* | NP | Tetraodontiformes | Mm1 | 2012-2020 | [1] |
| CVI |  | MZ934353 | *P. filosa* | NP | Tetraodontiformes | Mm1 | 2012-2020 | [1] |
| CVI |  | MZ934364 | *P. filosa* | NP | Carangiformes | Xg3 | 2012-2020 | [1] |
| CVII |  | MZ934354 | *P. filosa* | NP | Tetraodontiformes | Mm1 | 2012-2020 | [1] |
| CVIII |  | MZ934355 | *P. filosa* | NP | Tetraodontiformes | Mm1 | 2012-2020 | [1] |
| CIX |  | MZ934356 | *P. filosa* | NP | Tetraodontiformes | Mm* | 2012-2020 | [1] |
| CX |  | MZ934357 | *P. filosa* | NP | Tetraodontiformes | Mm* | 2012-2020 | [1] |
| CX |  | MZ934361 | *P. filosa* | NP | Tetraodontiformes | Mm* | 2012-2020 | [1] |
| CXI |  | MZ934358 | *P. filosa* | NP | Tetraodontiformes | Mm* | 2012-2020 | [1] |
| CXII |  | MZ934359 | *P. filosa* | NP | Tetraodontiformes | Mm* | 2012-2020 | [1] |
| CXIII |  | MZ934360 | *P. filosa* | NP | Tetraodontiformes | Mm* | 2012-2020 | [1] |
| CXIV |  | MZ934362 | *P. filosa* | NP | Tetraodontiformes | Mm* | 2012-2020 | [1] |
| CXV |  | MZ934363 | *Pennella* sp. | NP | Carangiformes | Nd1 | 2012-2020 | [1] |
| CXVI | IV / 4 | PP908442 | *P. filosa** | WM | Mysticeti | Bp2 | 2020 | [2] |
| CXVII | IV / 5 | MZ934340 | *P. filosa* | NP | Tetraodontiformes | Tr2 | 2012-2020 | [1] |
| CXVII | IV / 5 | PP908447 | *P. filosa** | WM | Carangiformes | Xg5 | 2019 | [2] |
| CXVIII | IV / 5 | MG701283 | *P. filosa** | WM | Tetraodontiformes | Mm | 2005 | [3] |
| CXIX | IV / 5 | MG701284 | *P. filosa** | WM | Tetraodontiformes | Mm | 2005 | [3] |
| CXX | IV / 5 | MG701288 | *P. filosa** | WM | Odontoceti | Sc | NA | [3] |
| CXXI | IV / 5 | MG701293 | *P. filosa** | WM | Odontoceti | Sc | NA | [3] |
| CXXII | IV / 4 | MZ934322 | *P. filosa* | NP | Carangiformes | Ch1 | 2012-2020 | [1] |
| CXXIII | IV / 5 | MZ934323 | *P. filosa* | NP | Carangiformes | Ka1 | 2012-2020 | [1] |
| CXXIV | IV / 5 | MZ934324 | *P. filosa* | NP | Carangiformes | Ka1 | 2012-2020 | [1] |
| CXXV | IV / 5 | MZ934325 | *P. benzi* | NP | Scombriformes | Lf1 | 2012-2020 | [1] |
| CXXVI | IV / 4 | MZ934326 | *P. filosa* | NP | Tetraodontiformes | Mm5 | 2012-2020 | [1] |
| CXXVI | IV / 4 | MZ934344 | *P. filosa* | NP | Carangiformes | Xg3 | 2012-2020 | [1] |
| CXXVII | IV / 5 | MZ934327 | *P. filosa* | NP | Tetraodontiformes | Mm* | 2012-2020 | [1] |
| CXXVIII | IV / 5 | MZ934328 | *P. filosa* | NP | Tetraodontiformes | Mm* | 2012-2020 | [1] |
| CXXIX | IV / 4 | MZ934329 | *P. filosa* | NP | Tetraodontiformes | Mm* | 2012-2020 | [1] |
| CXXIX | IV / 4 | MZ934331 | *P. filosa* | NP | Tetraodontiformes | Mm* | 2012-2020 | [1] |
| CXXX | IV / 4 | MZ934330 | *P. filosa* | NP | Tetraodontiformes | Mm* | 2012-2020 | [1] |
| CXXXI | IV / 4 | MZ934332 | *P. filosa* | NP | Tetraodontiformes | Mm* | 2012-2020 | [1] |
| CXXXII | IV / 4 | MZ934333 | *P. filosa* | NP | Tetraodontiformes | Mm* | 2012-2020 | [1] |
| CXXXIII | IV / 4 | MZ934334 | *P. filosa* | NP | Tetraodontiformes | Mm* | 2012-2020 | [1] |
| CXXXIV | IV / 4 | MZ934335 | *P. filosa* | NP | Tetraodontiformes | Mm* | 2012-2020 | [1] |
| CXXXIV | IV / 4 | MZ934337 | *P. filosa* | NP | Tetraodontiformes | Mm* | 2012-2020 | [1] |
| CXXXV | IV / 4 | MZ934336 | *P. filosa* | NP | Tetraodontiformes | Mm* | 2012-2020 | [1] |
| CXXXVI | IV / 4 | MZ934338 | *P. filosa* | NP | Tetraodontiformes | Mm* | 2012-2020 | [1] |
| CXXXVII | IV / 4 | MZ934339 | *P. filosa* | NP | Tetraodontiformes | Mm* | 2012-2020 | [1] |
| CXXXVIII | IV / 5 | MZ934341 | *P. filosa* | NP | Tetraodontiformes | Tr3 | 2012-2020 | [1] |
| CXXXIX | IV / 4 | MZ934342 | *P. instructa* | NP | Carangiformes | Xg1 | 2012-2020 | [1] |
| CXL | IV / 5 | MZ934343 | *P. instructa* | NP | Carangiformes | Xg1 | 2012-2020 | [1] |
| CXLI | IV / 4 | MZ934345 | *P. filosa* | NP | Carangiformes | Xg3 | 2012-2020 | [1] |
| CXLII | IV / 4 | MZ934346 | *P. filosa* | NP | Carangiformes | Xg3 | 2012-2020 | [1] |
| CXLIII | IV / 4 | MZ934347 | *P. filosa* | NP | Carangiformes | Xg3 | 2012-2020 | [1] |
| CXLIV | IV / 4 | MZ934348 | *P. filosa* | NP | Carangiformes | Xg3 | 2012-2020 | [1] |
| CXLV | IV / 4 | MZ934349 | *P. filosa* | NP | Carangiformes | Xg3 | 2012-2020 | [1] |

**^§^**Abbreviations for host species: *Bb*, sei whale (*Balaenoptera borealis*); *Be*, Bryde’s whale (*Balaenoptera edeni*); *Bp*, fin whale (*Balaenoptera physalus*); *Ch*, common dolphinfish (*Coryphaena hippurus*); *Cs*, Pacific saury (*Cololabis saira*); *Dd*, common dolphin (*Delphinus delphis*); *Gg*, Risso's dolphin (*Grampus griseus*); *Gm*, long-finned pilot whale (*Globicephala melas*); *Ka*, striped marlin (*Kajikia audax*); *Lf*, Escolar (*Lepidocybium flavobrunneum*); *Mn*, humpback whale (*Megaptera novaeangliae*); *Mm*, ocean sunfish (*Mola mola*); *Nd*, pilotfish (*Naucrates ductor*); *Pw*, North Pacific armorhead (*Pentaceros wheeleri*); *Sc*, striped dolphin (*Stenella coeruleoalba*); *Sq*, Japanese amberjack (*Seriola quinqueradiata*); *Tr*, Japanese pufferfish (*Takifugu rubripes*); *Xg*, swordfish (*Xiphias gladius*).

**Table S2.** Classification of host species of *Pennella* spp. according to their potential interoceanic connectivity (or lack of), inferred from data on genetic differentiation and migration between ocean basins in the literature. Abbreviations: *Atl*, Atlantic Ocean; *Ind*, Indian Ocean; *Med*, Mediterranean Sea; *Pac*, Pacific Ocean; *SH*, Southern Hemisphere. Within the two groups, host species are ordered by decreasing geographic range.

| **Common name** | **Scientific name** | **Geographic range** | **Interoceanic connectivity** | **Genetic structure and migration** |
| --- | --- | --- | --- | --- |
|  | | | | |
| Interoceanic connectivity | | | | |
| Humpback whale | *Megaptera novaeangliae* | Global | Med-Atl-Ind (Ind-Pac?) | Genetic differentiation between north Atl, north Pac, and SH (Ruegg et al., 2013), but Atl-Ind gene flow through South Africa (Kershaw et al., 2017) and no genetic differentiation between southeastern Ind and south Pac (Jackson et al., 2014); Atl visitors into Med (Russo et al., 2016) |
| Ocean sunfish | *Mola mola* | “ | Med-Atl, Ind-Pac | Genetic differentiation between Atl-Med and Ind-Pac subject to revision (Pope et al., 2010); Atl migrants into Med during winter (Sousa et al., 2016) |
| Swordfish | *Xiphias gladius* | Tropical-subpolar | Atl-Pac | Genetic differentiation between Med and Atl, but not between Atl and Pac (Kotoulas et al., 2007) |
| Sei whale | *Balaenoptera borealis* | Subtropical-subpolar, absent in Med | Atl-Pac | Genetic differentiation between north Atl, north Pac, and SH (Huijser et al., 2018; Taguchi et al., 2021), except one Atl individual with a north Pac haplotype (Huijser et al., 2018) |
| Striped dolphin | *Stenella coeruleoalba* | Tropical-temperate | Atl-Pac | Genetic differentiation between Med and Atl, and Med and Pac; but not between Atl and Pac (Bourret et al., 2007) |
| Common dolphin | *Delphinus delphis* | “ | Med-Atl | Genetic differentiation between Med and Atl, and within Med; some females move from Med into Atl (Natoli, 2008) |
|  |  |  |  |  |
| Interoceanic isolation/ regional range | | | | |
| Fin whale | *Balaenoptera physalus* | Temperate-polar |  | Genetic differentiation between Med and Atl (Notarbartolo‐di‐Sciara et al., 2003), and between north Atl and north Pac (Archer et al., 2019) |
| Risso’s dolphin | *Grampus griseus* | Tropical-temperate |  | Genetic differentiation between Med and Atl (Gaspari et al., 2007) |
| Mahi-mahi or common dolphinfish | *Coryphaena hippurus* | “ |  | Interoceanic migration restricted by cold water (Díaz-Jaimes et al., 2010); genetic differentiation between Med and Atl (Maggio et al., 2019) |
| Brydei’s whale | *Balaenoptera brydei* | Tropical-temperate, absent in Med |  | Genetic differentiation between north and south Pac, and eastern Ind (Kanda et al., 2007); lack of data from western Ind and Atl |
| Escolar | *Lepidocybium flavobrunneum* | “ |  | Genetic differentiation between Ind-Pac and Atl (Brendtro et al., 2008) |
| Long-finned pilot whale | *Globicephala melas* | North Atl (temperate-subpolar) and SH (subpolar) |  | Genetic differentiation between Med and Atl (Verborgh et al., 2016) |
| Striped marlin | *Kajikia audax* | Indopacific (tropical-temperate) |  | Genetic differentiation within Pac (Purcell and Edmands, 2011) |
| Pilot fish | *Naucrates ductor* | Circumtropical |  | Lack of genetic data between oceanic basins |
| North Pacific armorhead | *Pentaceros wheeleri* | North Pac |  |  |
| Pacific saury | *Cololabis saira* | “ |  |  |
| Japanese amberjack | *Seriola quinqueradiata* | Northwestern Pac |  |  |
| Japanese pufferfish | *Takifugu rubripes* | “ |  |  |

**Table S3.** Genetic diversity of *Pennella* across its two classes of hosts; including diversity between the superfamilies Mysticeti and Odontoceti, and five orders of teleosts. Values represent mean nucleotide sequence divergence (% K2P ± standard error) between (below diagonal) and within (shaded values on diagonal) host taxa.

| **Grouping** | **Host taxon** | **Mysticeti**  **(N= 35)** | **Odontoceti**  **(N= 7)** | **Teleostei**  **(N= 147)** |  |  |
| --- | --- | --- | --- | --- | --- | --- |
| **Class** | Cl. Mammalia |  |  |  |  |  |
|  | Spfam. Mysticeti | 2.15 ± 0.33 |  |  |  |  |
|  | Spfam. Odontoceti | 2.86 ± 0.43 | 3.06 ± 0.61 |  |  |  |
|  | Cl. Teleostei | 3.96 ± 0.52 | 4.54 ± 0.58 | 5.38 ± 0.67 |  |  |
|  |  |  |  |  |  |  |
| **Teleost order** |  | **Tetraodontiformes**  **(2 spp, N= 61)** | **Beloniformes**  **(1 sp, N= 36)** | **Carangiformes**  **(5 spp, N= 36)** | **Acropomatiformes**  **(1 sp, N= 4)** | **Scombriformes**  **(1 sp, N= 10)** |
|  | O. Tetraodontiformes | 6.79 ± 0.90 |  |  |  |  |
|  | O. Beloniformes | 4.99 ± 0.70 | 0.60 ± 0.12 |  |  |  |
|  | O. Carangiformes | 6.97 ± 0.88 | 5.05 ± 0.67 | 7.25 ± 0.87 |  |  |
|  | O. Acropomatiformes | 4.95 ± 0.71 | 0.54 ± 0.14 | 5.02 ± 0.68 | 0.45 ± 0.22 |  |
|  | O. Scombriformes | 4.96 ± 0.68 | 1.62 ± 0.44 | 4.99 ± 0.65 | 1.57 ± 0.46 | 1.12 ± 0.26 |

**Table S4.** Genetic diversity of *Pennella* across its two classes of hosts; including diversity between the superfamilies Mysticeti and Odontoceti, for two separate geographic regions. Values represent mean nucleotide sequence divergence (% K2P ± standard error) between (below diagonal) and within (shaded values on diagonal) host taxa.

| **Geographic region** | **Host taxon** | **Mysticeti** | **Odontoceti** | **Teleostei** |
| --- | --- | --- | --- | --- |
| **western Mediterranean** | Cl. Mammalia |  |  |  |
|  | Spfam. Mysticeti (N= 22) | 2.33 ± 0.37 |  |  |
|  | Spfam. Odontoceti (N= 7) | 2.99 ± 0.45 | 3.06 ± 0.60 |  |
|  | Cl. Teleostei (N= 6) | 6.05 ± 0.95 | 6.16 ± 0.95 | 6.63 ± 1.11 |
|  |  |  |  |  |
| **North Pacific** | Cl. Mammalia |  |  |  |
|  | Spfam. Mysticeti (N= 13) | 1.52 ± 0.34 |  |  |
|  | Cl. Teleostei (N= 141) | 3.71 ± 0.47 |  | 5.24 ± 0.68 |
|  |  |  |  |  |

**References in Supplementary Material**

Archer, FI, Brownell Jr, RL, Hancock-Hanser, BL, Morin, PA, Robertson, KM, Sherman, KK, Calambokidis, J, Urbán J, Rosel, PE, Mizroch, SA, Panigada, S and Taylor, BL (2019) Revision of fin whale *Balaenoptera physalus* (Linnaeus, 1758) subspecies using genetics. Journal of Mammalogy 100(5), 1653-1670.

Bourret, VJ, Macé, MR, and Crouau-Roy, B (2007) Genetic variation and population structure of western Mediterranean and northern Atlantic *Stenella coeruleoalba* populations inferred from microsatellite data. Journal of the Marine Biological Association of the United Kingdom 87(1), 265-269.

Brendtro, KS, McDowell, JR, and Graves, JE (2008) Population genetic structure of escolar (*Lepidocybium flavobrunneum*). Marine Biology 155, 11-22.

Díaz-Jaimes, P, Uribe-Alcocer, M, Rocha-Olivares, A, García-de-León, FJ, Nortmoon, P and Durand, JD (2010) Global phylogeography of the dolphinfish (*Coryphaena hippurus*): the influence of large effective population size and recent dispersal on the divergence of a marine pelagic cosmopolitan species. Molecular Phylogenetics and Evolution 57(3), 1209-1218.

Gaspari, S, Airoldi, S and Hoelzel, AR (2007) Risso’s dolphins (*Grampus griseus*) in UK waters are differentiated from a population in the Mediterranean Sea and genetically less diverse. Conservation Genetics 8, 727-732.

Huijser, LA, Bérubé, M, Cabrera, AA, Prieto, R, Silva, MA, Robbins, J, Kanda, N, Pastene, LA, Goto, M, Yoshida, H, Víkingsson, GA and Palsbøll, PJ (2018) Population structure of North Atlantic and North Pacific sei whales (*Balaenoptera borealis*) inferred from mitochondrial control region DNA sequences and microsatellite genotypes. Conservation Genetics 19, 1007-1024.

Jackson, JA, Steel, DJ, Beerli, P, Congdon, BC, Olavarría, C, Leslie, MS, Pomilla, C, Rosenbaum, H and Baker, CS (2014) Global diversity and oceanic divergence of humpback whales (*Megaptera novaeangliae*). Proceedings of the Royal Society B: Biological Sciences 281(1786), 20133222.

Kanda, N, Goto, M, Kato, H, McPhee, MV and Pastene, LA (2007) Population genetic structure of Bryde’s whales (*Balaenoptera brydei*) at the inter-oceanic and trans-equatorial levels. Conservation Genetics 8, 853-864.

Kershaw, F, Carvalho, I, Loo, J, Pomilla, C, Best, PB, Findlay, KP, Cerchio, S, Collins, T, Engel, M, Minton, G, Ersts, P, Barendse, J, Kotze, P, Razafindrakoto, Y, Ngouessono, S, Meÿer, M, Thornton, M and Rosenbaum, HC (2017) Multiple processes drive genetic structure of humpback whale (*Megaptera novaeangliae*) populations across spatial scales. Molecular Ecology 26(4), 977-994.

Kotoulas, G, Mejuto, J, Antoniou, A, Kasapidis, P, Tserpes, G, Piccinetti, C, Peristeraki, P, Oikonomaki, K and Magoulas, A (2007) Global genetic structure of swordfish (*Xiphias gladius*) as revealed by microsatellite DNA markers. Collective Volume of Scientific Papers ICCAT 61(1), 79-88.

Maggio, T, Allegra, A, Andaloro, F, Pedro Barreiros, J, Battaglia, P, Butler, CM, Cuttitta, A, Rodrigues Jorge Fontes, M, Freitas, R, Gatt, M, Karakulak, FS, Macias, D, Nicosia, A, Oxenford, HA, Saber, S, Rodrigues, NV, Yildiz, T and Sinopoli, M (2019) Historical separation and present-day structure of common dolphinfish (*Coryphaena hippurus*) populations in the Atlantic Ocean and Mediterranean Sea. ICES Journal of Marine Science 76(4), 1028-1038.

Notarbartolo‐di‐Sciara, G, Zanardelli, M, Jahoda, M, Panigada, S and Airoldi, S (2003) The fin whale *Balaenoptera physalus* (L. 1758) in the Mediterranean Sea. Mammal Review 33(2), 105-150.

Pope, EC, Hays, GC, Thys, TM, Doyle, TK, Sims, DW, Queiroz, N, Hobson, VJ, Kubicek, L and Houghton, JD (2010) The biology and ecology of the ocean sunfish *Mola mola*: a review of current knowledge and future research perspectives. Reviews in Fish Biology and Fisheries 20, 471-487.

Purcell, CM and Edmands, S (2011) Resolving the genetic structure of striped marlin, *Kajikia audax*, in the Pacific Ocean through spatial and temporal sampling of adult and immature fish. Canadian Journal of Fisheries and Aquatic Sciences 68(11), 1861-1875.

Ruegg, K, Rosenbaum, HC, Anderson, EC, Engel, M, Rothschild, A, Baker, CS and Palumbi, SR (2013) Long-term population size of the North Atlantic humpback whale within the context of worldwide population structure. Conservation Genetics 14, 103-114.

Russo, D, Sgammato, R and Bosso, L (2016) First sighting of the humpback whale *Megaptera novaeangliae* in the Tyrrhenian Sea and a mini-review of Mediterranean records. Hystrix 27(2), 219.

Sousa, LL, Queiroz, N, Mucientes, G, Humphries, NE and Sims, DW (2016) Environmental influence on the seasonal movements of satellite-tracked ocean sunfish *Mola mola* in the north-east Atlantic. Animal Biotelemetry 4(1), 1-19.

Suyama, S, Nakagami, M, Naya, M and Ueno, Y (2012) Migration route of Pacific saury *Cololabis saira* inferred from the otolith hyaline zone. Fisheries Science 78, 1179-1186.

Taguchi, M, Goto, M, Milmann, L, Siciliano, S, Tiedemann, R and Pastene, LA (2021) New insights into the genetic structure of sei whales (*Balaenoptera borealis*) at the inter-oceanic scale. Cetacean Population Studies 3, 152-163.

Verborgh, P, Gauffier, P, Esteban, R, Giménez, J, Cañadas, A, Salazar-Sierra, JM and de Stephanis, R (2016) Conservation status of long-finned pilot whales, *Globicephala melas*, in the Mediterranean Sea. Advances in Marine Biology 75, 173-203.
